# The natural alkaloid nitidine chloride targets RNA polymerase I to inhibit ribosome biogenesis and repress cancer cell growth

**DOI:** 10.1038/s41420-025-02396-x

**Published:** 2025-03-22

**Authors:** Igor Voukeng, Jing Chen, Denis L. J. Lafontaine

**Affiliations:** RNA Molecular Biology, Fonds de la Recherche Scientifique (F.R.S./FNRS), Université Libre de Bruxelles (ULB), Biopark Campus, Gosselies, Belgium

**Keywords:** RNA metabolism, Long non-coding RNAs, Organelles

## Abstract

Nature is an abundant and largely untapped source of potent bioactive molecules. Ribosome biogenesis modulators have proven effective in suppressing cancer cell growth and are currently being evaluated in clinical trials for anticancer therapies. In this study, we characterized the alkaloid nitidine chloride (NC), produced by the endemic Cameroonian plant *Fagara* (and other plants). We demonstrate that NC kills cancer cells regardless of their p53 status and inhibits tumor growth in vitro. Furthermore, NC profoundly suppresses global protein synthesis. Treatment of human cells with NC causes severe nucleolar disruption and inhibits pre-rRNA synthesis by destabilizing key factors required for recruitment of RNA polymerase I to ribosomal DNA promoters. In vitro, NC intercalates into DNA and inhibits topoisomerases I and II. Consistently, NC treatment activates a DNA damage response. We propose that the torsional stress on rDNA caused by topoisomerase inhibition leads to loss of RNA polymerase I function and to shutdown of ribosome biogenesis. Although NC has long been suspected of possessing anticancer properties, here we provide a molecular explanation for its mechanism of action. In budding yeast cells, interestingly, NC inhibits cell growth, impairs ribosome biogenesis, and disrupts nucleolar structure. This suggests that its mode of action is at least partially evolutionarily conserved.

## Introduction

Ribosomes are essential nanomachines in our cells, responsible for protein synthesis. Each ribosome is composed of two subunits of unequal size, with specialized functions in translation. The small subunit, 40S, reads the messenger RNA in the decoding site, while the large ribosomal subunit, 60S, assembles the amino acids in the peptidyl transferase center [1].

Ribosome biogenesis starts in the nucleolus, a multi-layered biomolecular condensate formed by liquid-liquid phase separation [2] and other phase transitions, with synthesis of a long precursor ribosomal RNA (rRNA) formed by RNA polymerase I. This long precursor encodes three of the four mature rRNAs, which are released by extensive processing [3].

Ribosome biogenesis is a key cellular process intimately linked to cell proliferation [4]. The quantity of ribosomes produced by any cell in the body must match its exact metabolic needs at any given time, and this requires exquisite regulation. Producing too many functional ribosomes may fuel tumorigenesis leading to cancer, whereas insufficient ribosome production characterizes disease states termed ribosomopathies [5]. Often deeply rooted in embryonic development, ribosomopathies are tissue-specific disorders affecting primarily the blood, brain, and bones [5].

Controlling ribosome production is becoming possible thanks to innovative therapeutic approaches [6, 7]. Notably, compounds that target RNA polymerase I, reducing its activity (eg. CX-5461 and BMH-21) [8–10], have proved able to kill cancer cells [11–16]. CX-5461 is currently engaged in clinical trials proving the concept that ribosome biogenesis modulators offer great potential in human therapeutics (see ClinicalTrials.gov).

Nature is a limitless source of potent bioactive molecules [17]. The properties of such compounds have been explored for centuries, all over the globe, in traditional medicine, but relatively few of them have been developed as modern drugs [18]. As potential producers of therapeutic molecules, it is important to consider species that might even be distant from us by billions of years of evolution. A remarkable example of this is *psilocybin*, a psychedelic compound produced by mushrooms. On human brain neuronal cells, this drug acts on receptors that the mushrooms have never encountered. By desynchronizing the brain [19], psilocybin has broad benefits in terms of mental health [20], notably in the treatment of severe depression, addictions, and post-traumatic stress disorder. This illustrates the need to preserve life diversity to explore natural resources sustainably.

Bioactive molecules of natural origin can potentially be extracted from any life form. Most explored so far are plants and marine organisms. Plant alkaloids, notably, form a very important family of phytochemicals to which we are exposed in our everyday lives. They include molecules as ordinary as caffeine and familiar drugs such as morphine, used for pain relief, and quinine, an anti-malarial. Marine natural products, especially ones from species living in extreme environments, are also particularly interesting because of the distinct elemental compositions and chemical scaffolds of some of them. Bioactive molecules have been extracted from organisms as diverse as algae, sponges, crustaceans, and tunicates, and the bioactivities they show are numerous. Some compounds, for example, are known to exert anti-cancer, anti-inflammatory, or neuroprotective effects [21, 22]. Twenty drugs derived from marine natural products have been approved by the FDA to treat cancer, chronic neuropathic pain, or cardiovascular disease, and >30 are in different phases of clinical trials [21, 22].

Being essential to protein biosynthesis, ribosomes are common targets of bioactive compounds, often produced by species competing with one another for access to an ecological niche. Many clinically relevant antibiotics target the ribosomes of disease-causing microbes, thus inhibiting translation. Other compounds specifically recognize human ribosomes. This is notably the case of the plant alkaloid haemanthamine (HAE), extracted from the daffodil flower (*Amaryllidaceae* family) [23]. HAE binds to the peptidyl transferase of the large ribosomal subunit to inhibit protein synthesis [23]. In addition, it inhibits ribosome biogenesis in the nucleolus, causing p53 stabilization in response to nucleolar stress activation [23]. Another interesting example is amodiaquine, an antimalarial drug, that also stabilizes p53 by nucleolar stress activation [24].

**Fig. 1:**
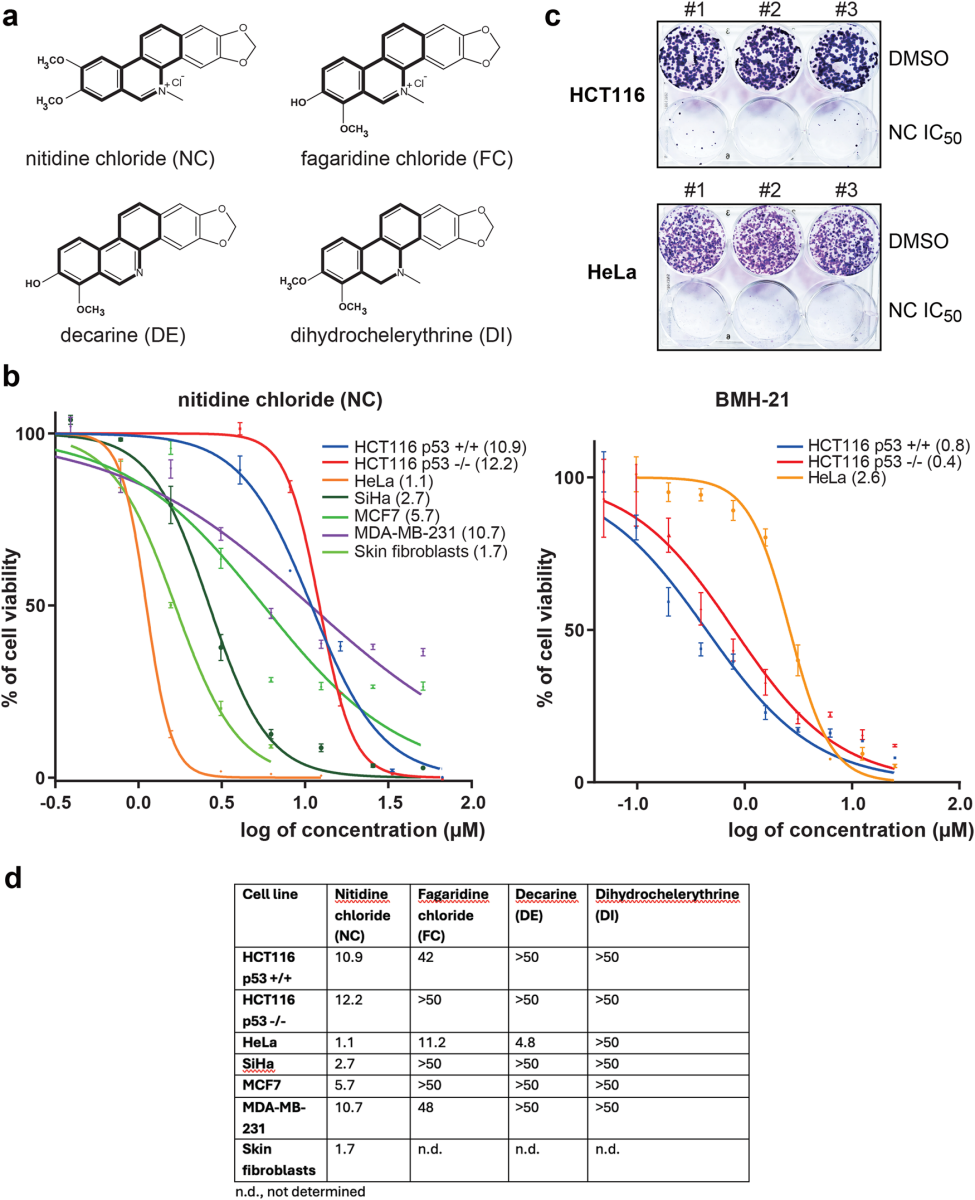
Nitidine chloride inhibits cancer cell proliferation and tumorigenesis in vitro. **a** Chemical structure of nitidine chloride (NC) and of three chemical congeners. The shared phenanthridine backbone is highlighted as a thick line. **b** Cell proliferation assay. The data are displayed as % cell viability *vs*. log concentration of drug (µM). Cell viability was assessed with a resazurin assay performed after 48 h of treatment. For each tested compound, seven concentrations were evaluated (0.4, 0.8, 1.6, 3.1, 6.2, 12.5, and 25 µg/ml), with each concentration tested in triplicate (see “Materials and Methods” for details). The IC_50_ calculated with GraphPad Prism 9 is indicated next to the cell line and in (**d**). **c** In vitro tumorigenesis assay. Soft agar colony formation assay, showing the number and size of colonies present 10 days after plating HeLa and HCT116 p53 +/+ cells treated or not with NC (respective IC_50_ concentrations used). Experiments performed in triplicate (#1 to #3). **d** IC_50_ (µM) of NC and congeners on cells of various origins.

In this work we have focused on *nitidine chloride* (NC) because of its suspected antitumor action [25–34]. NC is an alkaloid with a phenanthridine backbone (Fig. 1), extracted from several plants, including *Zanthoxylum nitidum* -also known as “Liang-Mian-Zhen” and frequently used in Chinese herbal medicine [35], *Zanthoxylum tessmannii* (or *Fagara tessmannii*, a *Cameroonian* plant), *Toddalia asiatica*, and *Evodia rutaecarpa* [36]. Besides its proposed anti-tumor properties, NC exhibits a remarkable range of additional biological activities: antimicrobial [37], antiviral and antifungal [38], antimalarial [39], anti-inflammatory [40], and antioxidant. Despite its promising therapeutic potential, and connections to several key cell signaling pathways controlling cell growth [25, 27, 28, 30–33, 41, 42], the precise molecular mechanism of action of NC has remained unknown [43].

Here we reveal that NC inhibits cancer cell proliferation and tumorigenesis in vitro through its protein-synthesis-downregulating capacity. Specifically, we show that the drug potently inhibits RNA polymerase I function by causing loss of metabolic stability of factors important for polymerase recruitment to rDNA promoters, leading to disruption of the nucleolus and loss of rRNA and ribosome production. We also demonstrate that NC is a DNA-intercalating agent that inhibits topoisomerases I and II in vitro, likely exerting its inhibitory effect on RNA polymerase I by inducing torsional stress in rDNA. Interestingly, we show that this compound also inhibits cell growth, suppresses ribosome biogenesis, and alters nucleolar structure in a distant ancestor of human cells: budding yeast. This indicates that its mode of action has been conserved at least partly through evolution.

## Results

### Nitidine chloride inhibits cancer cell proliferation and tumorigenesis in vitro

In a search for novel natural compounds with antiproliferative and potential anticancer effects, we focused on a natural benzophenanthridine alkaloid extracted from plants: *nitidine chloride*, because this class of compounds has shown promising anticancer effects [36, 44].

We used a classical resazurin assay to test the effect of NC on cell proliferation in six cancer cell lines and in primary cells (skin fibroblasts). In this assay, metabolically active cells reduce resazurin (blue) to resorufin (pink), which is assayed by fluorescence. In practice, cells were treated for 48 h with seven concentrations of NC (ranging from 25 to 0.4 µg/ml in 2-fold dilution steps). We selected cancer cells originating from different tissues: colon (HCT116), cervix (HeLa, SiHa), and breast (MCF7, MDA-MB-231). This selection includes cells expressing functional p53 (HCT116 p53 +/+, MCF7) or not (HCT116 p53 -/-, HeLa, SiHa, and MDA-MB-231) and representing distinct grades of the disease (MDA-MB-231 cells, which are triple negative, are more aggressive than MCF7 cells). For comparison and to investigate drug selectivity, we also tested three chemically related compounds: fagaridine chloride (FC), decarine (DE), and dihydrochelerythrine (DI).

NC emerged as the most potent inhibitor of cell proliferation, with an IC_50_ in the range of 1–10 µM (Fig. 1b and Fig. 1d). Although sharing the same phenanthridine backbone, the other tested compounds were mostly far less active, requiring doses >50 µM (except DE and FC on HeLa cells). For comparison, BMH-21 is active in the 1–2 µM range against three representative cancer cell lines (Fig. 1b), as previously described [9].

Close inspection of the effects of NC on cell proliferation revealed that its inhibitory effect is independent of cell p53 status. This was notably demonstrated by comparing HCT116 p53 +/+ and HCT116 -/- cells, which appeared similarly sensitive to the compound. Interestingly, the sensitivity towards NC of cells originating from the same tissue (breast) could vary up to two-fold; this may correlate with aggressiveness (more aggressive cells appearing to be more resistant). Also interesting was the observation that HeLa cells showed similar sensitivity to NC and BMH-21, whereas HCT116 cells, independently of their p53 status, were 10 times more sensitive to BMH-21 than to NC (Fig. 1b). Such differential sensitivity highlights the interest of developing additional cell-type-specific ribosome biogenesis modulators which, in addition to targeting ribosome biogenesis, may activate different regulatory loops (see “Discussion” and Fig. 8).

Because the two reference cell lines used throughout this work, HeLa and HCT116, exhibit a 10-fold difference in sensitivity to nitidine chloride, we will henceforth, to facilitate comparisons, systematically refer to their respective ½ IC_50_, 1× IC_50_, and 2× IC_50_ values.

Next we performed a clonogenicity assay with both HCT116 and HeLa cells, treating them in triplicate (#1 to #3) with NC at their corresponding IC_50_. NC was found to massively inhibit tumorigenesis in vitro, as hardly any colonies were counted upon drug treatment (Fig. 1c).

In conclusion, NC displays potent antiproliferative activity on multiple cancer cells of various origins and on primary dermal fibroblasts. In addition, NC inhibits tumorigenesis in vitro in both HCT116 and HeLa cells.

### Nitidine chloride inhibits protein synthesis

**Fig. 2:**
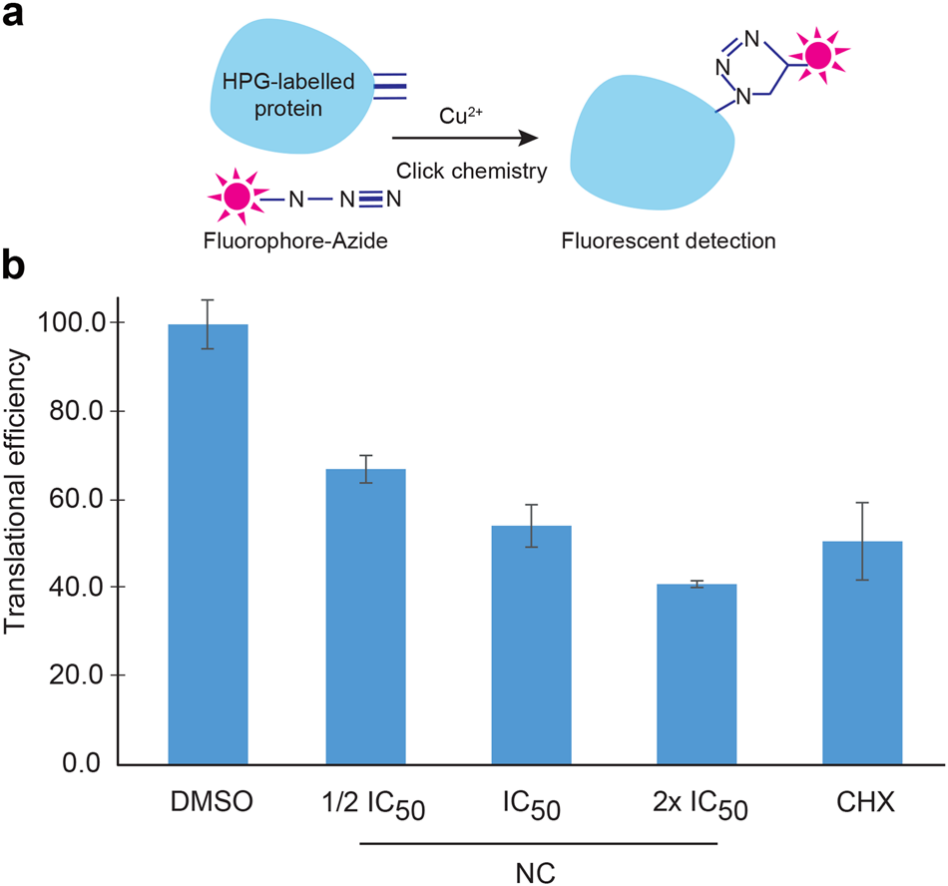
Nitidine chloride inhibits global protein synthesis. **a** Principle of detection of newly synthesized proteins by click chemistry. A covalent link is established between L-homopropargylglycine (HPG)-labeled proteins and fluorophore-azide in the presence of copper. Fluorescently labeled proteins are detected by FACS. **b** HeLa cells treated with NC at ½ IC_50_, IC_50_, and 2x IC_50_ for 24 h, labeled with HPG 1 h prior to collection, and analyzed by FACS to measure amino-acid incorporation into proteins. As controls, cells were treated with vehicle alone (DMSO 0.1%), or with cycloheximide (CHX, 5 µM), a well-known translational inhibitor. The analysis was done in triplicate. The same number of viable cells (4000) was analyzed in each condition. The data are expressed as translation efficiencies (signal normalized to control).

To start to understand at the molecular level how NC inhibits cell proliferation, we tested potential effects on protein synthesis. We used metabolic labeling to assess global translation. This was done by incorporating a methionine analog (L-homopropargylglycine, HPG) into nascent proteins and clicking a fluorescent group onto it for quantitative detection by FACS (Fig. 2a).

In this experiment we used HeLa cells, which are particularly sensitive to NC (Fig. 1b and Fig. 1d), and treated them for 24 h at three different drug concentrations: half the IC_50_, the IC_50_, and twice the IC_50_. Cells were labeled with HPG 1 h prior to collection (see “Materials and Methods”). Treating cells with NC led to a substantial reduction of translation: ~50% at IC_50_ (Fig. 2b), comparable to that observed upon treatment with the well-known translational inhibitor cycloheximide used as control [45]. NC-mediated inhibition of translation appeared dose-dependent, which suggests specificity.

**Fig. 3:**
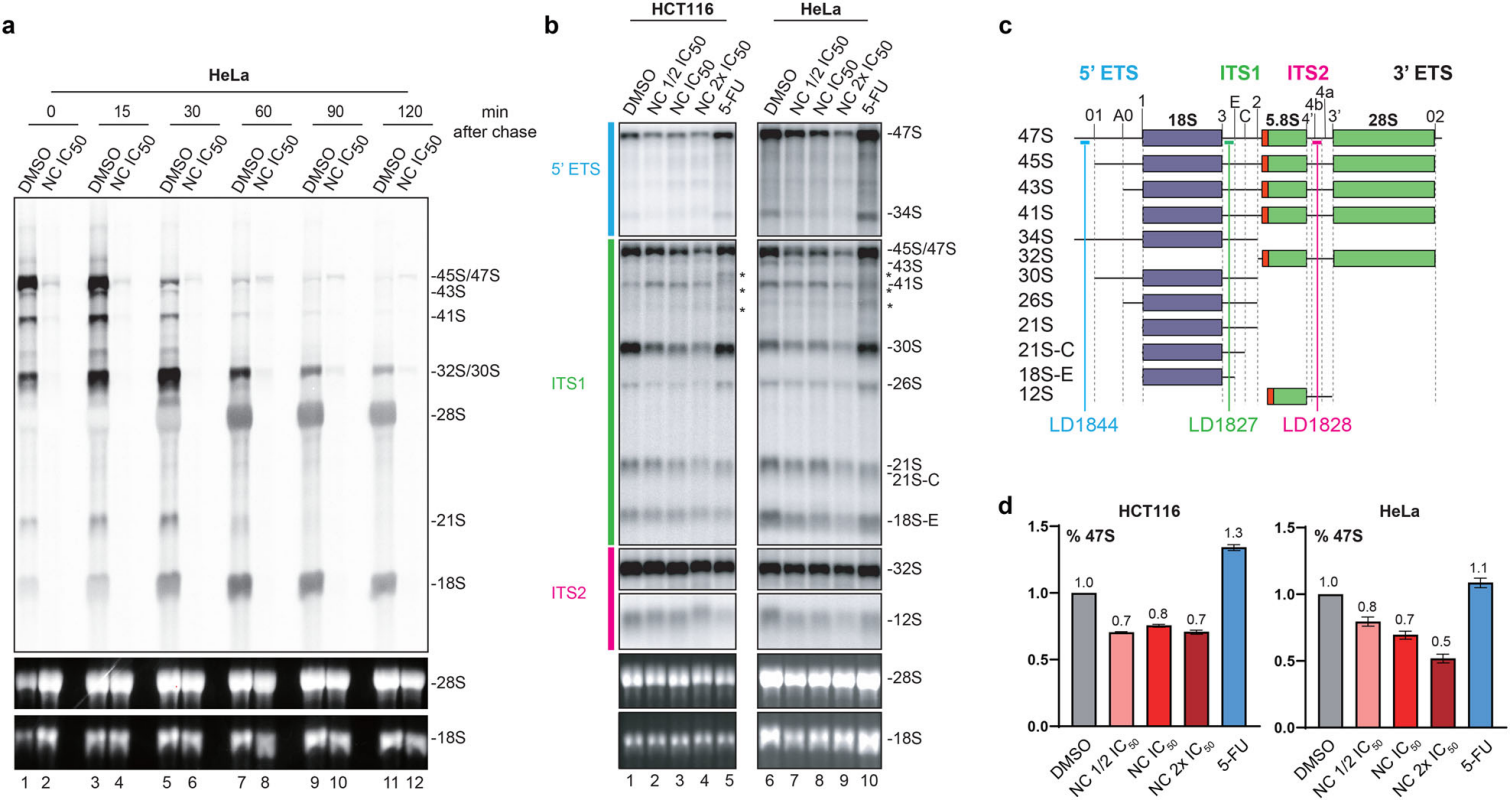
Nitidine chloride inhibits ribosome biogenesis. **a** Pre-rRNA processing dynamics, analyzed by pulse-chase labeling. Hela cells treated with NC at their IC_50_ for 5 h were incubated with L-(methyl-^3^H)-methionine for 30 min, chased with an excess of cold methionine, and collected at different time points over a 2 h period. Total RNA was extracted, resolved on an agarose gel, and transferred onto a GeneScreen membrane to which a tritium-sensitive plate was subsequently exposed. The ethidium bromide-stained agarose gel revealing steady-state levels of mature 18S and 28S rRNAs is shown to illustrate even loading. Vehicle control, 0.1% DMSO. **b** Steady-state analysis of pre-rRNA processing by northern blotting. Five micrograms of total RNA extracted from HeLa or HCT116 cells treated for 24 h with the indicated concentration of NC (½ IC_50_, IC_50_, or 2 x IC_50_ of the cell line concerned) was resolved on a denaturing agarose gel, transferred to a nylon membrane, and probed with specific radioactively labeled oligonucleotides to detect major pre-rRNA species. As a control we used 5-FU (15 µM), which inhibits early pre-rRNA processing steps and thus causes accumulation of 47S. Vehicle control, 0.1% DMSO. **c** Structure of the primary transcript (47S) synthesized by RNA polymerase I, positions of the probes (LD1844, LD1827, and LD1828) used in panel b, and precursor rRNAs detected. **d** Phosphor imager quantification of the 47S primary transcript, showing clear reduction upon NC treatment. Data normalized to the DMSO control. All experiments performed in triplicate.

In conclusion, NC inhibits global protein synthesis in a dose-dependent fashion. As discussed below, ribosome biogenesis is repressed by NC treatment. Quantifying the levels of mature rRNAs at the time point of analysis of global translation (Fig. 2) revealed a ~20–30% reduction of mature 18S and 28S rRNAs (see Fig. 3b), which appears compatible with the observed reduction of protein synthesis.

### Nitidine chloride inhibits ribosome biogenesis

Having observed this drastic effect of NC on protein synthesis, we next wondered if ribosome biogenesis might be affected by the drug.

In eukaryotes, three out of four mature rRNAs, the 18S, 5.8S, and 28S rRNAs, are produced through extensive processing of a single long polycistronic precursor (47S) synthesized by RNA polymerase I in the nucleolus (see Fig. 3c).

We first performed in vivo metabolic labeling. HeLa cells treated or not for 5 h with NC at IC_50_ concentration were pulsed-labeled with L-(methyl-^3^H)-methionine for 30 min, chased with excess cold methionine, and collected at different time points over a 2-h period. Total RNA was extracted, resolved on a denaturing agarose gel, transferred to a membrane, and processed for fluorographic detection (Fig. 3a).

In control cells (DMSO), the primary transcript (47S) was easily detected. It was found to convert rapidly into the different successive pre-rRNA precursors, with eventual production of mature 18S and 28S rRNAs. As expected, the amount of a precursor species (for example, 32S or 21S) decreased as the corresponding product (respectively 28S or 18S) was formed (Fig. 3a).

In NC-treated cells, the amount of primary transcript produced was severely reduced (compare the amounts of 45S/47S in NC-treated and DMSO-treated cells at time point 0), and hardly any cleaved products were detected. This indicates that RNA synthesis was severely impaired by the drug (Fig. 3a). In addition, the low level of primary transcripts formed during the pulse remained stable over time: it was higher at the last time point of the assay, 120 min., in the drug-treated sample than in the control. This reveals that, in addition to inhibiting RNA synthesis, NC also inhibits the early steps of pre-rRNA processing.

Next, we used northern blotting to analyze steady-state pre-rRNA processing in more detail (Fig. 3b). For this analysis, we used both HCT116 and HeLa cells. Total RNA extracted from cells treated for 24 h at their respective ½ IC_50_, IC_50_, or 2x IC_50_ concentrations of NC was resolved on denaturing agarose gels and processed for northern blotting with radioactively labeled probes designed to detect the abundant pre-rRNA precursors (Fig. 3c).

Again, the most striking effect observed in both cell lines, upon treating cells with NC, was the substantially reduced steady-state level of 47S primary transcript (Fig. 3b and see panel d for quantification). Note that the amount of primary transcript may appear higher in Fig. 3b than in Fig. 3a. This is simply because in Fig. 3b all transcripts are detected by probing, whereas in Fig. 3a only newly synthesized transcripts (“nascent transcripts”) are labeled. In HCT116 and HeLa cells treated at the IC_50_ concentration, the residual amounts of 47S were respectively ~80% and ~70%. This reduction of 47S was accompanied by a decrease in most pre-rRNA intermediates detected, including 30S, 26S, 21S, 21S-C, 18S-E, and 12S. The 34S RNA, resulting from direct cleavage of the primary transcript in the internal transcribed spacer 1 (ITS1), was also decreased. Such a decrease of most detected intermediates is compatible with an inhibition of RNA synthesis, as concluded from the pulse-chase assay (Fig. 3a).

Although pre-rRNA synthesis is massively suppressed upon NC treatment, as demonstrated by the extremely low residual levels of nascent transcripts directly radioactively labeled in cells (Fig. 3a), it may be surprising that some pre-rRNA species appear to accumulate stably in the steady-state analysis (northern blotting) presented in Fig. 3b. Notably, this is the case of 32S (ITS2 probe panel), which appears less affected than other intermediates such as 30S (ITS1 probe panel). This is simply because each pre-rRNA species has its own half-life, determined by a complex combination of factors, including how rapidly it is processed, its level of RNA folding and packaging with ribosomal proteins and assembly factors (offering protection from salient RNases), and whether it is subject to surveillance and degradation.

As a control, cells were treated with 5-FU, an anticancer drug used clinically and recently shown to alter ribosome function [46], in addition to its known effects on pre-rRNA processing [23]. In contrast to NC, 5-FU had a stronger effect on the early steps of pre-rRNA processing than on rRNA synthesis, since we observed high steady-state levels of both primary transcript (47S) and 34S. In addition, unlike NC, 5-FU treatment led to production of multiple truncated precursor forms (labeled with stars). HeLa and HCT116 cells responded similarly to each of the tested drugs.

In conclusion, NC strongly inhibits ribosome biogenesis at both the pre-rRNA synthesis and initial processing steps, thus affecting translation and cell proliferation.

### Nitidine chloride disrupts nucleolar organization

The nucleolus is a multiphase biomolecular condensate whose morphology reflects its function [2]. It consists of three main nested layers: the inner fibrillar center (FC), the middle dense fibrillar component (DFC), and the outer granular component (GC). FC/DFC forming modules present in multiple copies in a single GC.

**Fig. 4:**
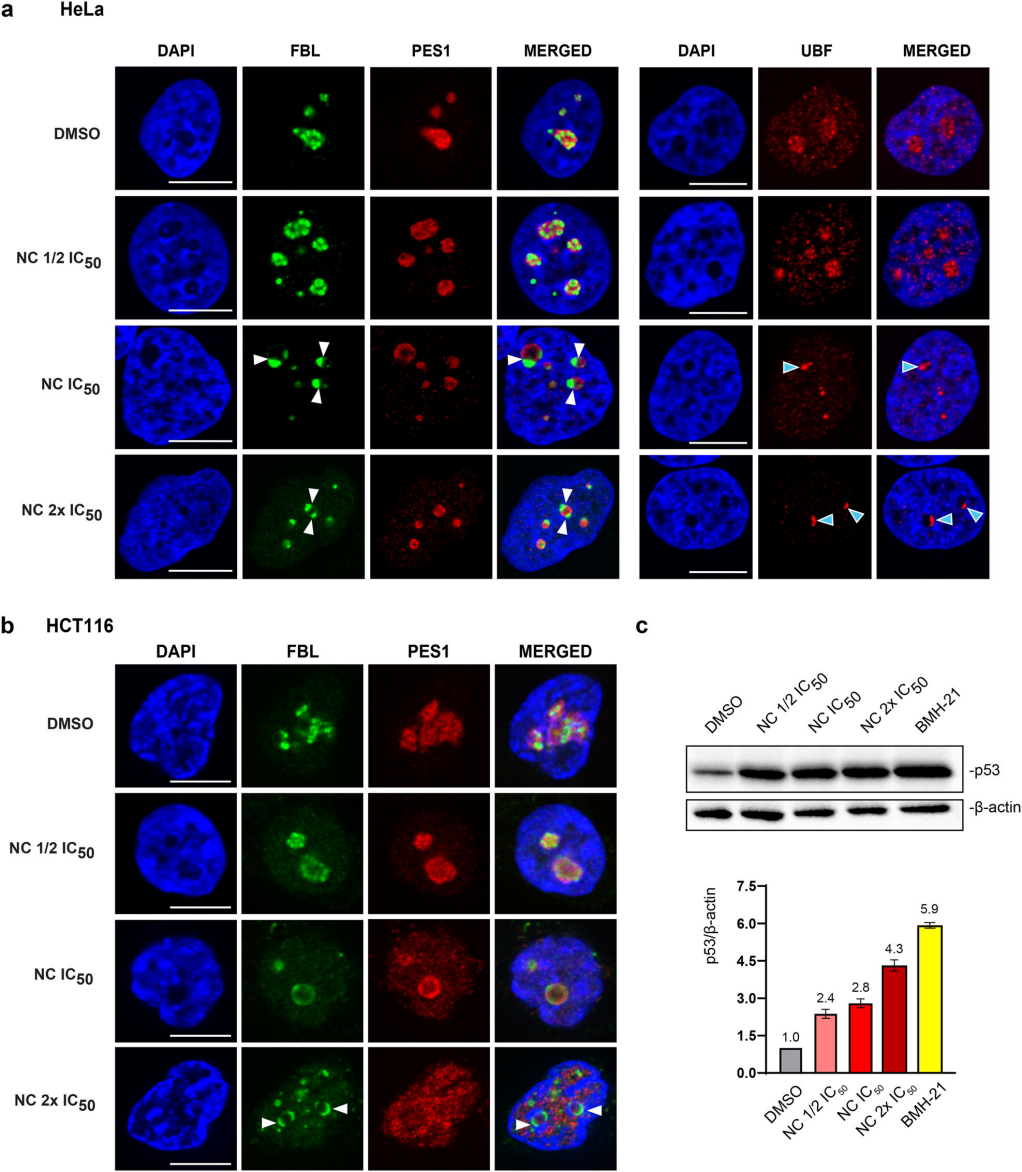
Nitidine chloride disrupts nucleolar organization and activates nucleolar surveillance. **a** HeLa cells treated with NC for 6 h at ½ IC_50_, IC_50_, or 2x IC_50_, were immunostained with antibodies specific to UBF, fibrillarin (FBL), or PES1. DAPI was used to stain DNA. White arrowheads indicate FBL caps; cyan arrowheads point to UBF foci. Scale bar, 10 µm. See Fig. S2 for expanded views. **b** HCT116 cells treated for 24 h with NC at ½ IC_50_, IC_50_, or 2x IC_50_were immunostained with antibodies specific to fibrillarin (FBL) or PES1. DAPI was used to stain DNA. Arrowheads indicate FBL caps. Scale bar, 10 µm. See Fig. S2 for expanded views. **c** Nucleolar surveillance is activated upon NC treatment. Top, Western blot evaluation of p53 amounts in HCT116 p53 +/+ cells treated for 24 h with NC at ½ IC_50_, IC_50_, or 2x IC_50_. As a control, cells were treated with BMH-21, known to elicit potent nucleolar surveillance activation (used at IC_50_ for 24 h). Bottom, ChemiDoc quantification of signals shown above. Three independent experiments were performed (*n* = 3). In all panels, DMSO was used at 0.1% as vehicle control. All experiments performed in triplicate.

In HeLa cells treated with different concentrations of NC, each layer of the nucleolus was detected by immunostaining with specific antibodies (Fig. 4a-b). Detection of the upstream binding transcription factor (UBF) was used to visualize the FCs, fibrillarin (FBL) was employed to highlight the DFCs, and pescadillo ribosomal biogenesis factor 1 (PES1) to reveal the GC. In control cells, all three proteins displayed the expected nested arrangement (Fig. 4a): the FBL stain was comprised within the PES1 staining (left panels, see DMSO/merged) and UBF displayed a discrete punctuate pattern at the core of the nucleolus (right panels).

In cells treated with NC at the IC_50_ concentration and above, fibrillarin formed distinctive “caps” abutted to PES1 (Fig. 4a, white arrowheads point to the interface between caps and PES1 signal), a phenotype known as nucleolar segregation and associated with RNA polymerase I inhibition (discussed in Ref. [2]). In parallel, UBF adopted a more compact and spatially restricted distribution (cyan arrowheads). Structural alterations of the nucleolus were also observed in HCT116 cells, including formation of fibrillarin “caps” and important release of PES1 into the nucleoplasmic space (Fig. 4b). Note that the treatment time was 6 h for HeLa cells and 24 h for HCT116 cells, which are more resistant to the drug (Fig. 1d). In both HeLa and HCT116 cells, the penetrance of the nucleolar disruption phenotype was nearly 100%, as shown in the expanded views displaying more cells (Fig. S2).

Previously we reported a method, known as iNo scoring [47, 48], for the quantitative and qualitative assessment of nucleolar disruption phenotypes. This method utilizes a series of discriminant shape and textural features to group similar phenotypes in a 2-D space by means of principal component analysis (PCA). We demonstrated that such clustering offers predictive insights into disrupted nucleolar function. One example involved the ribosomal proteins uL5 (RPL11) and uL18 (RPL5), which are assembled simultaneously during late-stage maturation of the large ribosomal subunit (their role in nucleolar surveillance is further illustrated in Fig. 4c). We showed that removing either uL5 or uL18 from cells causes nucleolar disruptions that appear highly similar. Remarkably, these treatments were grouped together in the PCA (see Fig. S3, group VII, and [47, 48]).

Applying the same scoring method to HeLa cells treated with NC at ½x IC_50_, IC_50_, and 2x IC_50_—alongside nine treatments known to affect nucleolar morphology to varying degrees and appropriate controls - revealed that at IC_50_ and 2x IC_50_, NC clustered with RNA polymerase I inhibitors causing cap formation (Fig. S3, group IV). Notably, each dot in the PCA represents at least 200 cells (and often more), with each treatment performed in triplicate. The systematic grouping of replicate treatments attests to the robustness of the analysis. Interestingly, at the low NC concentration of ½x IC_50_, where caps were not observed (Fig. 4a), the treatment did not group with group IV but instead formed an independent, stand-alone group (group II).

In conclusion, using a statistically validated image analysis pipeline (the iNo scoring method), we found NC to cluster functionally with compounds that inhibit RNA polymerase I, including two topoisomerase inhibitors: etoposide and camptothecin (see below, Fig. 6).

An important role of the antitumor protein p53 is to trigger a transcriptional program leading to elimination of problematic cells. In functional cells, p53 is maintained at a low level by constitutive ubiquitination, performed by Hdm2, and subsequent proteasomal degradation. When cells do not make ribosomes well, as when RNA polymerase I is inhibited, specific ribosomal components accumulate. These include the ribosomal proteins uL5 (RPL11) and uL18 (RPL5), which together with the 5S rRNA (independently produced by RNA polymerase III) form a stable trimeric complex that captures and sequesters Hdm2 away from p53. Under such conditions, p53 is stable and can exert its regulatory role in killing dysfunctional cells. This regulatory loop is known as ‘nucleolar surveillance’ (refs. [47, 49, 50]).

Since pre-rRNA synthesis is downregulated in NC-treated HCT116 cells (see Fig. 3a, b) and since HCT116 p53 +/+ cells express p53 normally, we examined whether NC treatment of these cells might lead to activation of nucleolar surveillance. In agreement with the above regulatory model, the answer was clearly yes: the level of p53 was increased ~3-fold upon drug treatment (Fig. 4c). In cells exposed to the known rRNA synthesis inhibitor BMH-21 (ref. [9]), used as a control, p53 was also stabilized. Thus, although sensitivity to NC does not strictly depend on the presence of functional p53 (See Fig. 1), this protein, when expressed, is stabilized upon exposure to the drug, and this stabilization likely contributes to the capacity of NC to inhibit cell proliferation in those circumstances.

In conclusion, treating cells with NC triggers a profound rearrangement of the nucleolus, often referred to as “nucleolar segregation” or nucleolar “caps”, a hallmark of RNA polymerase I inhibition. This is in agreement with the RNA analysis (Fig. 3).

### Nitidine chloride impacts factors important for rRNA synthesis

The rRNA synthesis inhibitor BMH-21 has been shown to promote proteasome-mediated degradation of RPA194, a subunit of RNA polymerase I (ref. [9]). This prompted us to test whether NC might impact the steady-state levels of factors important for rRNA synthesis.

**Fig. 5:**
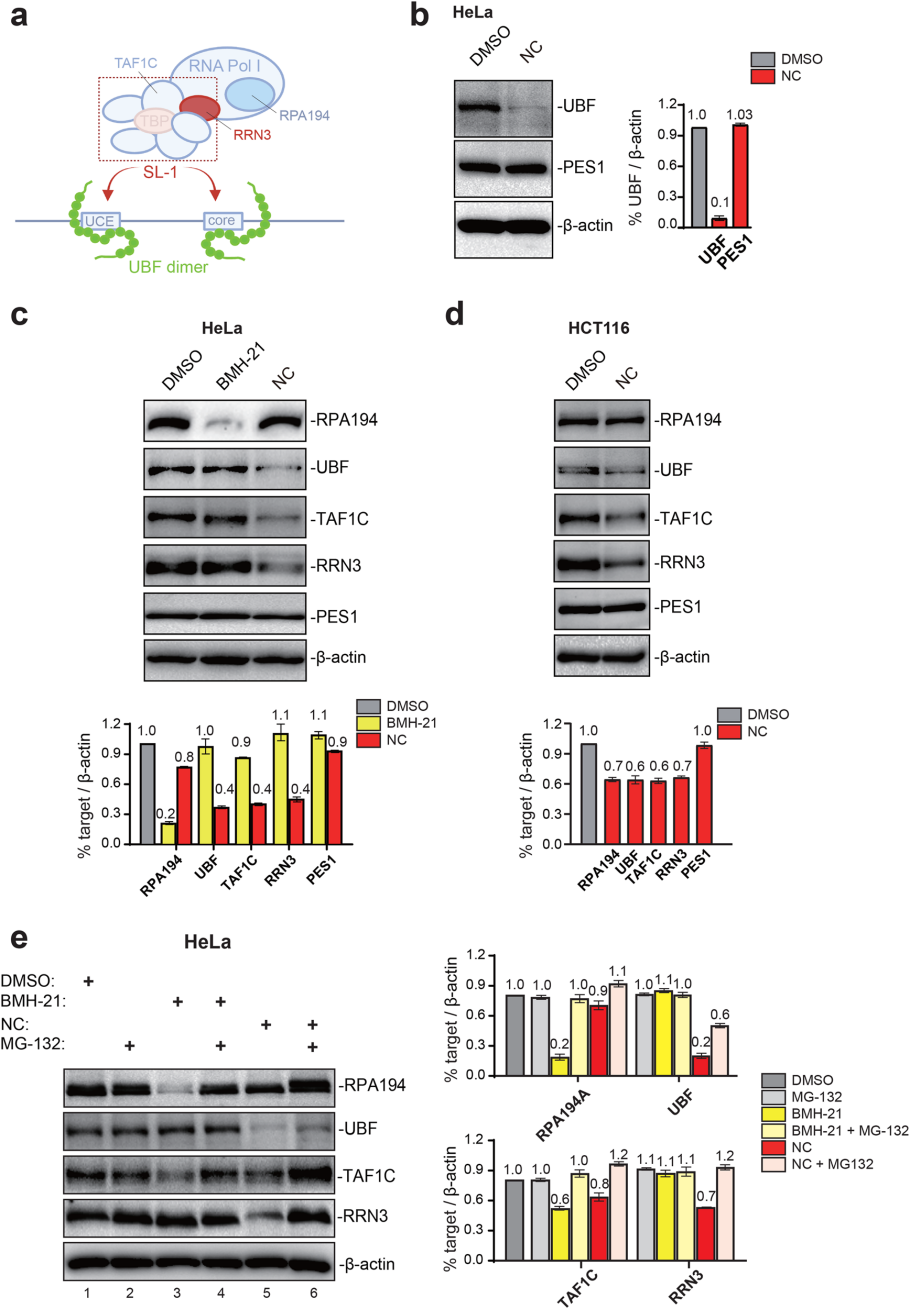
Nitidine chloride reduces the metabolic stability of RNA Polymerase I and the levels of factors important for its recruitment to the rDNA promoter. **a** Simplified description of the rDNA promoter and factors important for rRNA synthesis. Binding of the upstream binding factor (UBF) to the rDNA promoter (at upstream control elements, UCEs, and at the core element) is followed by recruitment of multisubunit selectivity factor 1 (SL-1). A stable UBF-SL-1 complex then recruits initiation-competent Pol I, containing RRN3. RRN3 constitutes a “bridge” between Pol I and SL-1. The initiation factor SL-1 consists of the TATA box-binding protein (TBP) and of several TATA box-associated factors (TAFs), including TAF1C. Adapted from ref. [51]. **b**–**d** Evaluation of steady-state levels of RNA Pol I and associated factors by western blotting in cells treated with NC. HeLa cells treated for 6 h. HCT116 cells treated for 24 h. In each case, specific antibodies were used for detection. As loading controls, PES1 and β-actin were used. Quantifications are presented (*n* = 3). **e** The metabolic stability of Pol I (RPA194), UBF, TAF1C, and RRN3 is affected by NC. HeLa cells treated for 24 h with the indicated drug in the presence or absence of a proteasome inhibitor (MG-132). Quantifications presented (*n* = 3). In all panels: NC, 2x IC_50_ of the cell line concerned; BMH-21, IC_50_; DMSO, 0.1%.

We first tested whether the drug might affect the upstream binding factor (UBF), because this factor plays a crucial role during pre-initiation complex formation (for details, see legend of Fig. 5a). In HeLa cells treated with NC for 6 h, UBF was substantially reduced (Fig. 5b). This reduction was highly specific, since another nucleolar protein, PES1 (used above to visualize the GC compartment of the nucleolus), was not affected.

Once bound to the rDNA promoter, UBF recruits the multi-subunit selectivity factor SL-1 and together they associate with RRN3-bound polymerase I to initiate pre-rRNA synthesis [51]. RRN3 acts as a sort of interface between SL-1 and Pol I. Having shown that UBF is severely affected by NC treatment, we investigated its effect on other components of the machinery. We assessed the amounts of key subunits of individual macromolecular complexes: TAF1C for SL-1 and RPA194 for Pol I; we also tested the interface protein RRN3 (see Fig. 5a). For UBF, TAF1C, and RRN3, we detected a substantial ~60% reduction (Fig. 5c, see quantifications). By comparison, Pol I (RPA194) was reduced by only ~20% (Fig. 5c).

HCT116 cells are less sensitive to NC than HeLa cells (Fig. 1), so they were exposed to the compound for 24 h as in the above-described nucleolar morphology analysis (see Fig. 4). Under these conditions, some reduction of UBF, TAF1C, and RRN3 was observed (Fig. 5d). The effects were never as strong as in HeLa cells but they were specific, as judged by the largely unaffected PES1 and β-actin levels.

Previous work on BMH-21 has indicated that it acts by affecting the metabolic stability of factors important for rRNA synthesis [9]. To see is this applies also to NC, we treated HeLa cells with NC in the presence and absence of the proteasome inhibitor MG-132 (Fig. 5e). When proteasome activity was inhibited in NC-treated cells, RPA194, TAF1C, and RRN3 were restored to their control levels (Fig. 5e, compare lanes 5 and 6 and see quantifications); UBF was also increased but did not reach its control level. This indicates that NC affects the metabolic stability of Pol I and of several of its cofactors. Note that BMH-21 impacted strongly the stability of RPA194 and to a lesser extent that of TAF1C (Fig. 5, lane 3), and that both factors were restored to control levels upon proteasomal inhibition (Fig. 5, lane 4). Unlike NC, BMH-21 did not impact the stability of UBF and RRN3. This indicates different modes of action.

In conclusion, NC severely impacts the steady-state levels of factors important for Pol I function, including to some extent that of Pol I itself and principally of factors important for its recruitment to rDNA promoters (UBF, TAF1C, and RRN3). This provides a molecular explanation of the effects of NC on protein synthesis, ribosome biogenesis, and nucleolar structure.

### Nitidine chloride is a DNA-intercalating agent that inhibits topoisomerases I and II and induces DNA damage

**Fig. 6:**
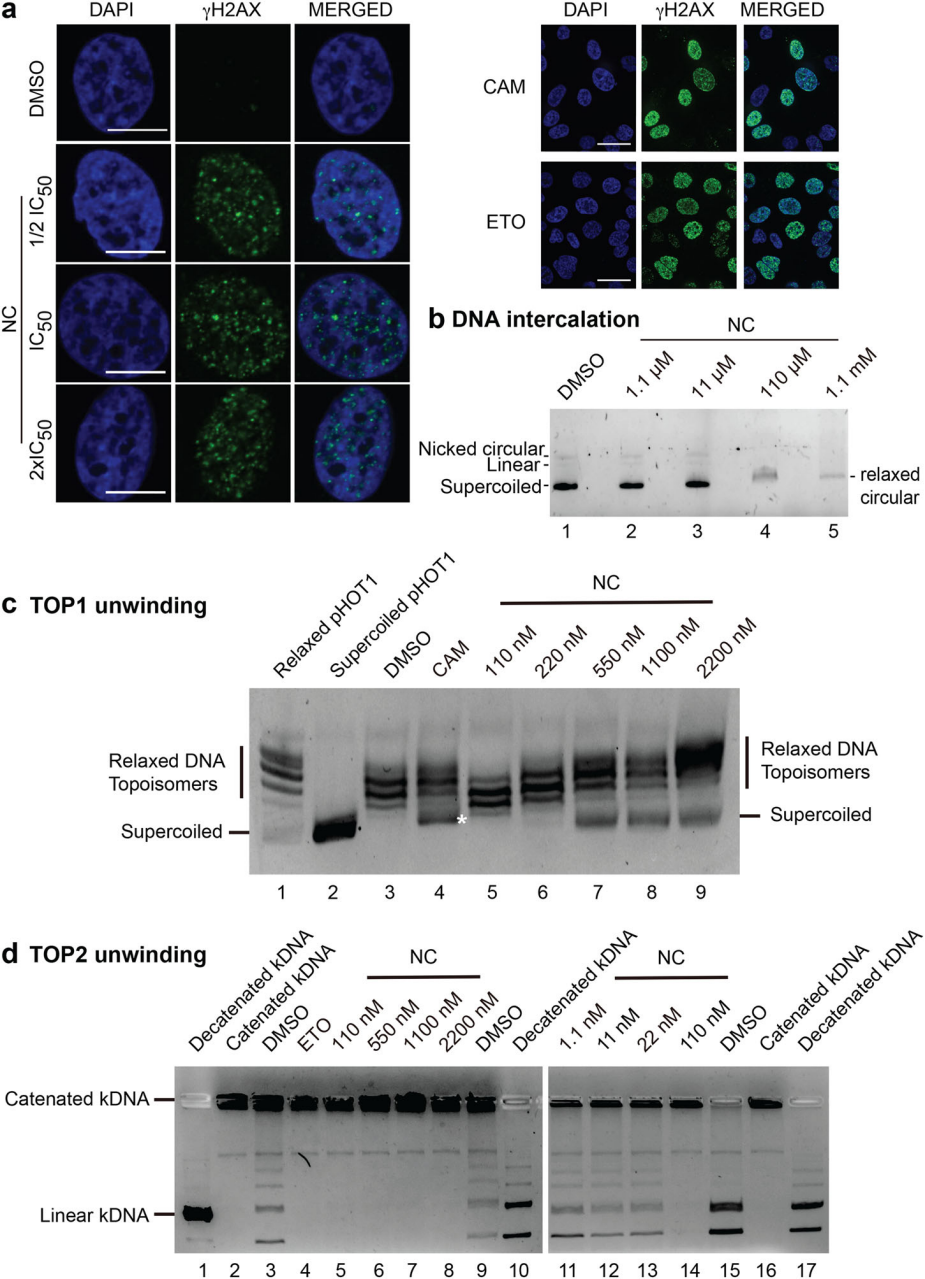
Effects of nitidine chloride on DNA metabolism. **a** NC activates DNA damage. Left, To assess the DNA damage response, HeLa cells were exposed for 1 h to NC at ½ IC_50_, IC_50_, or 2x IC_50_, immunostained for γ-H2AX, and imaged by fluorescence microscopy. DNA was stained with DAPI. 63x magnification, spin disk confocal. Scale bar, 10 µm. Right, as a control, cells were exposed to camptothecin (CAM, 1 µM) or etoposide (ETO, 10 µM) for 1 h. Vehicle control, 0.1% DMSO. Scale bar, 50 µm. **b** NC is a DNA-intercalating agent. Supercoiled pUC19 DNA was incubated with 0.2 µg/ml ethidium bromide (a concentration that does not shift supercoiled DNA to a relaxed circular form, see Fig. S4 for details) in the presence or absence of NC at increasing concentration. At concentration of NC 110 µM and above, the DNA was shifted and less intensely stained. This demonstrates that NC is a DNA-intercalating agent. DMSO was used as control. **c** NC is a TOP1 inhibitor. The conversion of supercoiled pHOT1 DNA, incubated in HeLa extracts at 37 °C for 30 min, to relaxed DNA topoisomers was used to monitor TOP1 activity (lane 3, 0.1% DMSO used as control). TOP1 activity was inhibited by addition of campthotecin (CAM, 5 µM, lane 4) leading to accumulation of supercoiled DNA (white star). To test if NC inhibits TOP1, a range of concentrations was tested (lanes 5–9). At concentration 220 nM and above, supercoiled DNA was detected, indicating TOP1 inhibition. Molecular weight markers for relaxed and supercoiled forms of pHOT1 are provided (lanes 1 and 2, respectively). **d** NC is a TOP2 inhibitor. The conversion of catenated kDNA, incubated in HeLa extracts at 37 °C for 30 min, to decatenated kDNA was used to monitor TOP2 activity (lane 3, 9, and 15, 0.1% DMSO used as control). TOP2 activity was inhibited by addition of etoposide (ETO, 50 µM, lane 4), abolishing conversion of catenated kDNA. To test if NC inhibits TOP2, a range of concentrations was tested (lanes 5–8 and lanes 11–14). At concentration 110 nM and above, only catenated kDNA was observed. This indicates TOP2 inhibition. Molecular weight markers for decatenated (lanes 1, 10, and 17) and catenated kDNA (lanes 2 and 16) are provided. All assays were performed in triplicate (*n* = 3).

The ribosome biogenesis modulator CX-5461 is an rDNA G4 quadruplex binder known to cause DNA torsional stress leading to DNA damage response activation [52]. It also acts as a topoisomerase II poison [53, 54]. DNA damage is typically monitored by detection of γH2AX foci. This prompted us to test whether NC might also lead to formation of γH2AX foci. We found this to be the case (Fig. 6a, left). As controls, cells were treated with topoisomerase inhibitors, camptothecin (CAM) or etoposide (ETO). In both cases this led to formation of abundant γH2AX foci (Fig. 6a, right).

On the basis of the observed DNA damage response elicited upon treatment of cells with NC, we were interested in learning if the drug acts as a DNA-intercalating agent.

Ethidium bromide (EthBr) is well known to intercalate into DNA, making it visible under UV light after gel electrophoresis. This is typically the case when supercoiled DNA, such as pUC19, is incubated with low concentrations of EthBr (e.g., 0.1 or 0.2 µg/ml, see Fig. S4a, lanes 1–2). Increasing the concentration of EthBr to 0.5 µg/ml unwinds the DNA, causing relaxation of the supercoiled state into a slower migrating relaxed circular form (Fig. S4, lane 3).

To test if NC is a DNA-intercalating agent, we incubated DNA with a low concentration of EthBr (0.2 µg/ml) that does not relax the DNA and then added increasing amounts of NC. Our question was: do NC and EthBr have a synergistic effect on DNA relaxation? We conclude that they do (Fig. 6b). Not only did addition of NC to EthBr-bound DNA prompt relaxation (Fig. 6b, lanes 4–5), but it also decreased the intensity of EthBr staining. This decrease indicates that EthBr was “competed off” by NC. Performing the reciprocal assay, i.e., first incubating the DNA with various concentrations of NC and then adding EthBr for visualization under UV light, led to the same conclusions: we observed less intensely stained, slower-migrating relaxed circular DNA (Fig. S4c). We conclude that NC is a DNA-intercalating agent.

Having shown that NC intercalates into DNA, we wondered if it might interfere with the function of topoisomerases by impacting DNA topology. Specifically, we used dedicated in vitro assays to test whether NC inhibits topoisomerase I (TOP1) and/or topoisomerase II (TOP2) (Fig. 6c, d).

For TOP1, we monitored the conversion of supercoiled DNA (pHOT1) to relaxed topoisomers (Fig. 6c, see “Materials and Methods”). When supercoiled pHOT1 DNA was incubated in purified HeLa extracts in the presence of DMSO used as a negative control, it was completely converted to relaxed topoisomers thanks to the presence of active TOP1 in the extract (Fig. 6c, lane 3). Note that molecular weight markers for relaxed topoisomers and supercoiled DNA are provided (see Fig. 6c, lanes 1 and 2, respectively). When camptothecin (CAM), a well-known inhibitor of TOP1, was added to the reaction, the conversion was incomplete and residual amounts of supercoiled DNA were observed (lane 4, white star). When increasing amounts of NC were added to the reaction (concentrations ranging from 110 to 2200 nM were tested), there was progressive accumulation of supercoiled DNA, the effects starting to be seen at 220 nM and becoming obvious at 550 nM and above. We conclude that NC is a potent TOP1 inhibitor.

For TOP2, we assessed decatenation of a kinetoplast DNA (kDNA) (Fig. 6d, see “Materials and Methods”). When catenated kDNA was incubated in purified HeLa extracts in the presence of DMSO used as a negative control, it was converted to decatenated forms thanks to the presence of active TOP2 in the extract (Fig. 6d, lanes 3, 9, and 15). Note that molecular weight markers for decatenated kDNA (Fig. 6d, lanes 1, 10, and 17) and catenated kDNA (Fig. 6d, lanes 2 and 16) are provided. When etoposide (ETO), a well-known inhibitor of TOP2, was added to the reaction, the decatenation of kDNA was abolished, and only catenated DNA was detected (Fig. 6d, lane 4). When increasing amounts of NC were added to the reaction (initially, concentrations ranging from 110 to 2200 nM were tested), decatenation was also completely inhibited (Fig. 6d, lanes 5–8). These observations prompted us to test a second series of lower concentrations of NC (ranging from 1.1 to 110 nM, Fig. 6d, lanes 11–14). This revealed progressive inhibition of decatenation, with marked effects starting at 110 nM. We conclude that NC is a potent TOP2 inhibitor.

In conclusion, NC is a potent inhibitor of TOP1 and TOP2 in vitro.

### NC mildly activates the integrated stress response

Ultimately, ribosome biogenesis inhibition will shut down translation. We reasoned that under such adverse conditions cells might adapt by rapidly reducing energy-intensive processes to enhance survival. Typically, the integrated stress response (ISR) is an adaptive pathway that temporarily mitigates cell stress by slowing protein production, promoting repair, and activating survival mechanisms [55]. During ISR, the translational factor eIF2α is phosphorylated, reducing translation and serving as a marker of ISR activation.

Total protein extracted from HeLa cells treated for 6 h with NC (2.2 µM), thapsigargin (Thaps, 0.5 µM, used as a control of ISR activation [56]), or DMSO (0.1%, used as negative control) were analyzed by western blotting with specific antibodies against unphosphorylated and phosphorylated eIF2α. As loading control, β-actin was used.

We observed a 2.3-fold accumulation of the phosphorylated form of eIF2α after NC treatment. This accumulation appears limited by comparison with the 8.8-fold increase in phospho-eIF2α triggered by Thaps treatment (Fig. S5a-b). Thaps treatment also led to formation of remarkable structures in the cytoplasm (Fig. S5c, white arrow), which were not detected upon NC treatment.

In conclusion, in addition to inhibiting ribosome biogenesis in the nucleolus, NC mildly activates ISR in the cytoplasm, rapidly inhibiting translation.

### Nitidine chloride inhibits ribosome biogenesis in yeast

Although ribosome biogenesis has become more complex in the course of eukaryotic evolution [57], aspects of this process are conserved and modulators originally described in human cells, such as BMH-21, have been shown to function also in yeast [58].

**Fig. 7:**
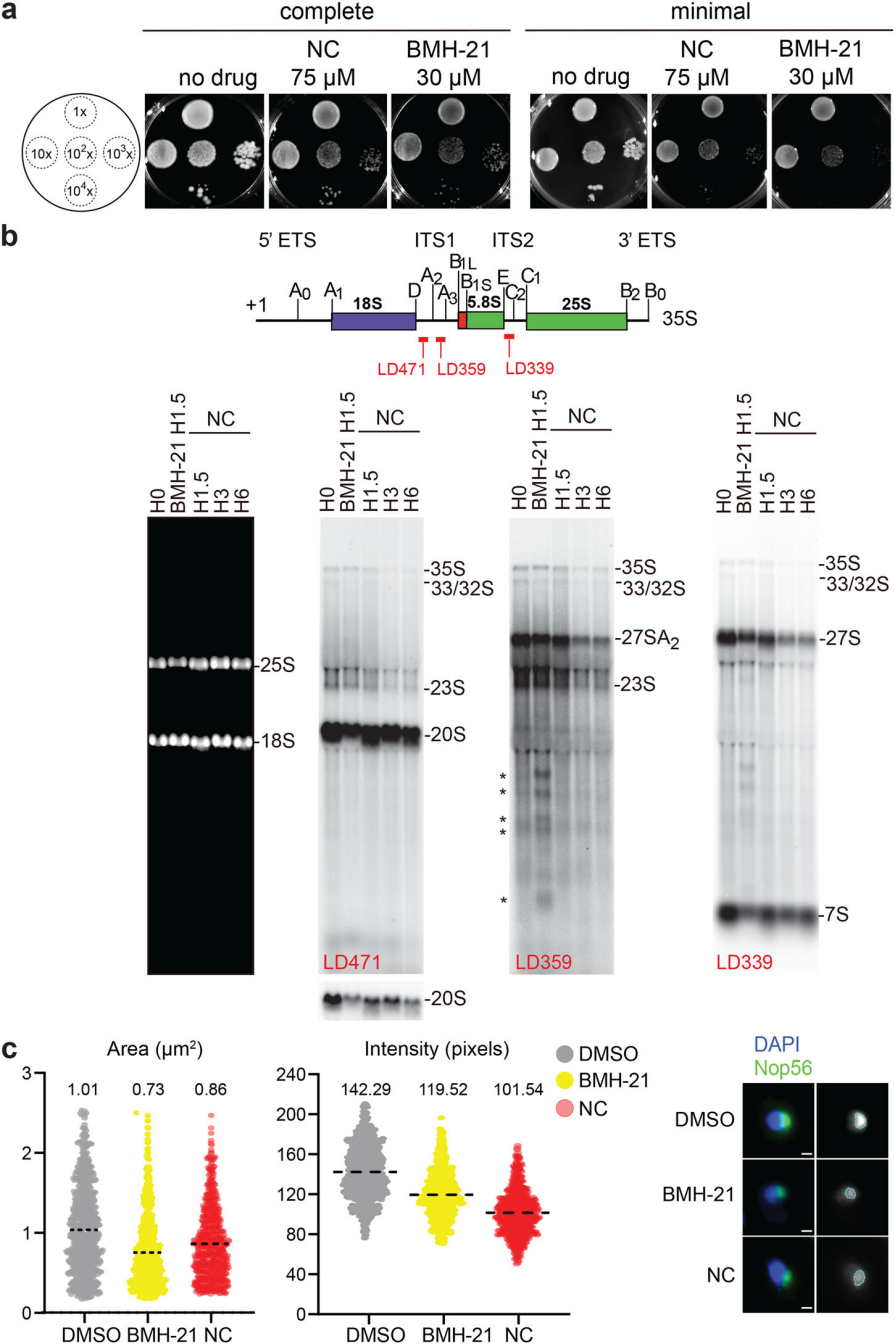
Nitidine chloride inhibits ribosome biogenesis in yeast. **a** Drop assay. A reference wild-type yeast strain (BY4741, Euroscarf) in suspension at OD_600_ = 0.5 was subjected to serial 10-fold dilutions and 10 µl of each dilution was spotted onto plates of complete or minimal medium containing the specified concentration of NC or BMH-21. The plates were incubated at 30 °C for 3 days. **b** Pre-rRNA processing analysis. Top, Schematic representing the structure of the yeast primary transcript (35S) synthesized by RNA polymerase I with sequences encoding the 18S, 5.8S and 25S rRNAs, the non-coding spacers (the 5’ and 3’ external transcribed spacers (ETS) and internal transcribed spacers (ITS) 1 and 2), and the positions of the probes used (LD339, LD359, and LD471) in the northern blots. Bottom, Total RNA (8 µg) extracted from yeast cells treated with 50 µM BMH-21 or 75 µM NC for the indicated time. The RNA was resolved on a denaturing agarose gel, transferred to a nylon membrane, and probed with specific radioactively labeled oligonucleotides to detect major pre-rRNA species. **c** Nucleolar area and intensity analysis. The nucleolus detected by fluorescence imaging in a yeast strain expressing endogenously GFP-tagged Nop56 was imaged by spinning disk confocal microscopy at 100× magnification and segmented by intensity-based thresholding with Image J. The area and intensity were extracted and computed. Numbers of cells analyzed were: 674, 581, and 646 for DMSO, BMH-21, and NC, respectively. Cells were treated for 2 h with 30 µg/ml BMH-21, or 75 µg/ml NC. All experiments performed in triplicate.

We therefore wondered if NC might inhibit yeast cell growth. Using a drop assay, we showed that it does, on both complete and minimal medium (Fig. 7a). An effect was seen at 30 µM NC and higher (here illustrated at 75 µM). As a control, yeast cells were also tested on BMH-21-containing plates, and this confirmed the previously described inhibition [58].

The effects of NC on ribosome biogenesis in yeast cells were tested by northern blotting (Fig. 7b). Steady-state levels of the primary transcript (35S) and of processing products (27S, 27SA_2_, etc.) were all diminished. This is compatible with reduced rRNA synthesis. Treatment of cells with BMH-21 led to accumulation of aberrant cleaved products (*), which were not seen upon incubation with NC.

The nucleolar area and intensity were quantified and shown to be reduced by 15% and 29%, respectively, as compared to isogenic wild-type cells (Fig. 7c). BMH-21 treatment showed essentially the same effects, albeit less marked than with NC.

In conclusion, NC appears to be active also in yeast cells, where it inhibits cell growth and ribosome biogenesis and impacts the structure of the nucleolus. This suggests at least partial evolutionary conservation of its mode of action.

## Discussion

Cancer cells rely on excessive protein production to fuel their unrestricted growth [59]. Ribosome biogenesis has emerged as a novel target for anticancer interventions [60–63]. Ribosome biogenesis modulators targeting the first step of the process, RNA synthesis, have entered clinical trials. By opening new therapeutic avenues, this has created a momentum to identify and characterize novel ribosome biogenesis modulators, ideally offering a range of different characteristics to broaden future therapeutic options. It is hoped, notably, that novel modulators will have differential effects on cells of different origin and distinct molecular mechanisms of action, and that they will elicit different cell stress responses and different regulatory loops.

Here we have focused on the natural alkaloid nitidine chloride, which has shown promising potential for human health but whose precise mechanism of action has remained unclear [36, 44].

In human cells, we confirm the inhibitory effect of nitidine chloride on cell proliferation. For this we have used a range of cancer cell lines and primary dermal fibroblasts (Fig. 1). We reveal that the compound strongly inhibits global protein synthesis (Fig. 2) and that this reflects its capacity to substantially downregulate rRNA synthesis. We have reached this conclusion biochemically, using metabolic labeling and northern blotting to demonstrate strong reduction of primary transcript synthesis and of production of all processed precursors (Fig. 3). At the cell biology level, we have used fluorescence microscopy to show dramatic reorganization of the nucleolar subphases, leading to segregation and formation of caps (Fig. 4 and Fig. S2), a phenotype consistent with RNA polymerase I inhibition. In a fine quantitative and qualitative analysis using the iNo scoring method [47, 48], based on a combination of discriminant shape and textural features of the nucleolus, NC was found to group with compounds, including topoisomerase inhibitors, that inhibit rRNA synthesis and cause formation of nucleolar caps (Fig. S3, group IV in PCA).

Nitidine chloride, an alkaloid extracted from plants, has a distinctive phenanthridine backbone. In our study it was interesting to test three closely related congeners which share the same biochemical backbone: fagarine chloride, decarine, and dihydrochelerythrine (Fig. 1a). Despite their molecular resemblance, all three were far less potent than nitidine chloride. This indicates that the peripheral chemical groups of the phenanthridine backbone confer strong specificity to the effects recorded (Fig. 1d).

We report that cancer cells originating from different tissues (breast, cervix, and colon) are differentially sensitive to nitidine chloride, with IC_50_ values in the 1–10 µM range (Fig. 1d). Notably, cervix cancer cells (HeLa) are 10 times more sensitive than colon cancer cells (HCT116). Interestingly, while HeLa cells show similar sensitivity to nitidine chloride and BMH-21, HCT116 cells are more sensitive to BMH-21 (Fig. 1b). Such differences in sensitivity between cells of different tissue origin highlight the need to identify and develop novel ribosome biogenesis modulators.

A founding principle underlying the use of ribosome biogenesis inhibitors in cancer therapy is that cancer cells are more sensitive to reduction of ribosome production and protein synthesis than non-cancerous ones. Potent downregulation of ribosomes and proteins should already be enough to kill cells, but ribosome biogenesis inhibitors additionally activate multiple cell death regulatory loops and may differ in the signaling pathways they trigger. In p53-positive cells, an important contributor to cell killing is nucleolar surveillance, activated via a mechanism involving Hdm2 titration by uL5-uL18-5S. As nucleolar surveillance is a p53-dependent pathway, one would expect strategies relying on its activation to be effective only against p53-positive cancers, i.e., roughly ~50% of all cancers. In actual fact, however, it has been shown for CX-5461 and BMH-21, the two best-studied cases so far, that they also kill p53-negative cells [9, 10]. In the case of CX-5461, this is notably because it induces a p53-independent DNA damage response in addition to nucleolar surveillance [52, 64–66].

*What about nitidine chloride?* In agreement with the nucleolar surveillance model, NC causes p53 stabilization in HCT116 p53 +/+ cells (Fig. 4c), but clearly, the drug can kill p53-negative cells as efficiently as it kills cells expressing p53 (Fig. 1). As in the case of other compounds of this class, this is due to inhibition of ribosome production and to a propensity also to activate DNA damage (Fig. 6a). We have shown that NC is a DNA-intercalating agent (Fig. 6b and Fig. S4) that inhibits topoisomerases I and II in vitro (Fig. 6c, d). We propose that the torsional stress induced by NC binding to DNA inhibits topoisomerases, leading to activation of a DNA damage response.

CX-5461 has been suggested to activate DNA damage by binding to G-quadruplex (G4) motifs [52] (which are particularly abundant in rDNA promoters), inducing torsional stress, and poisoning topoisomerase II (refs [53, 54]). As NC is predicted to have high affinity for G-rich sequences [67], a similar principle might apply to it as well.

The nucleolus is a multiphase biomolecular condensate whose nested layered organization is profoundly rearranged upon NC treatment: the inner core of the condensate, where the tandem arrays of rDNA lie, is exposed at the periphery of the disrupted organelle. This spatial topology, with rDNA exposed to the nucleoplasm, makes it particularly prone to DNA repair by making it accessible to repair factors normally excluded from nucleoli.

The effects of nitidine chloride on rRNA synthesis suggested to us that the metabolic stability of RNA polymerase I and/or its cofactors might be affected. This turned out to be the case. Molecularly, we show that the compound strongly impacts steady-state levels of proteins important for recruitment of RNA polymerase I to the rDNA promoter. These include UBF, the SL-1 component TAF1C, RRN3, and to some extent the largest subunit of the RNA polymerase itself (Fig. 5). We propose that intercalation of NC into DNA leads to displacement of rDNA-bound RNA Pol I transcription factors (including UBF, RRN3, and TAF1C). In combination with NC-induced torsional stress on ribosomal DNA, this could promote RNA polymerase I complex disassembly and explain loss of metabolic stability.

HCT116 cells, interestingly, show lesser reduction of UBF, TAF1C, and RRN3 than HeLa cells (Fig. 5). This may explain why the latter are 10 times more sensitive to the drug than the former (Fig. 1). We have proved that the affected process is protein stability, rather than protein synthesis, since inhibition of the proteasome by MG-132 treatment restored levels of most tested protein factors: RPA194, TAF1C, RRN3, and to a lesser extent UBF (Fig. 5e). For comparison, BMH-21 strongly impacts the stability of Pol I but not detectably that of cofactors, apart from TAF1C, which also displayed some reduction (Fig. 5e). Like those of NC, the destabilizing effects of BMH-21 were suppressed by proteasomal inhibition.

Nitidine chloride is a natural phytochemical used in folk medicine in Africa and China. We show here that NC is active on human cells. The fact that some organisms produce bioactive compounds that inhibit cell growth implies that those organisms have evolved resistance mechanisms, e.g., specific detoxification strategies or increased efflux [68]. As ribosome biogenesis is well conserved overall in eukaryotes, we wondered if species other than human might be affected. We found budding yeast to be sensitive to NC (Fig. 7). Furthermore, the observation that in budding yeast NC affects cell growth, rRNA synthesis, and nucleolar activity (as judged by the area and intensity of a nucleolar marker, the box C/D-associated protein NOP56 [69]), indicates that the mode of action of nitidine chloride has been at least partially conserved through evolution.

Previously, we have reported that the alkaloid haemanthamine, extracted from the bulbs of daffodils, inhibits ribosome biogenesis and ribosome function and activates nucleolar surveillance [23]. Although haemanthamine and nitidine chloride do not share the same chemical backbone (crinine-type versus benzophenantridine-type), they are both alkaloids extracted from plants and having specific effects on ribosome biogenesis. This fascinating family of molecules thus holds true promise for targeting dysregulated ribosome biogenesis in cancer.

**Fig. 8:**
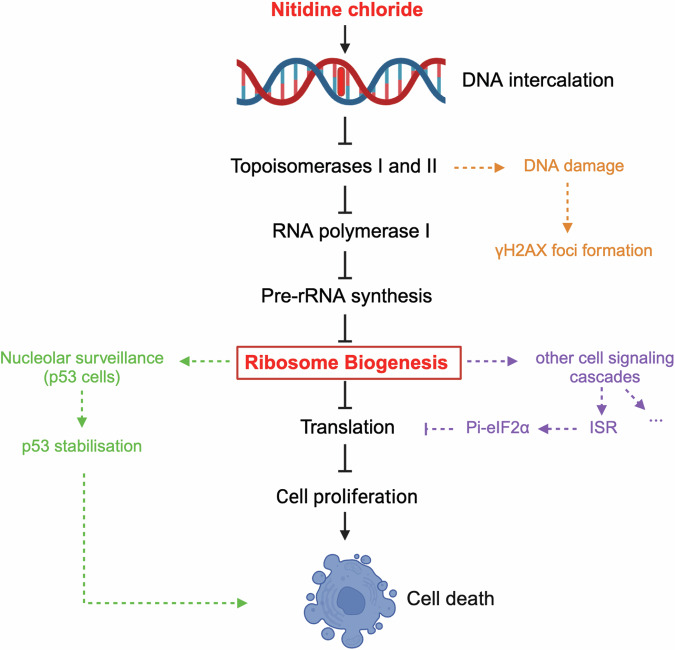
Model for the mechanisms of action of nitidine chloride. Nitidine chloride (NC) is a DNA-intercalating agent that binds DNA in vitro (Fig. 6b), inducing torsional stress and causing inhibition of topoisomerases I and II in vitro (Fig. 6c, d). Inhibition of topoisomerases may result in suppression of RNA polymerase I activity, pre-rRNA synthesis, and ribosome biogenesis (Figs. 3–5**)**. The shutdown of ribosome biogenesis suppresses global translation (Fig. 2), thereby inhibiting cell proliferation and inducing cell death (Fig. 1). In addition, several surveillance pathways may be activated upon NC treatment: (1) Inhibition of topoisomerases may activate a DNA damage response (formation of γH2AX foci, Fig. 6a). (2) Ribosome assembly inhibition may activate nucleolar surveillance (unassembled uL5-uL18-5S complex captures Hdm2, making it unable to modify p53, which escapes proteasomal degradation), causing p53 stabilization and cell death (Fig. 4c). (3) The integrated stress response may be activated as an attempt at survival by further repressing translation temporarily through eiF2α phosphorylation (Fig. S5). (4) Additional regulatory loops are likely to be activated (See “Introduction”).

In conclusion, we provide significant molecular insights into the effects of the natural alkaloid nitidine chloride on cell proliferation (Fig. 8). Nitidine chloride is a DNA-intercalating agent that induces rDNA torsional stress and topoisomerase inhibition, resulting in RNA polymerase I inhibition, suppression of rRNA production, nucleolar disruption, and subsequent downregulation of protein synthesis (Fig. 8). Additionally, several regulatory loops may be activated. In p53-positive cells, this includes nucleolar surveillance, leading to p53 stabilization (Fig. 4c), which contributes to cell death (Fig. 8). It also includes the integrated stress response, a survival pathway resulting in phosphorylation of eiF2α (Fig. S5), which contributes to acute protein synthesis inhibition (Fig. 8).

Nature has probed the chemical space for billions of years, producing an immense diversity of compounds whose properties remain largely unknown. Remarkably, compounds produced by one species can have beneficial or adverse effects on others, even ones that are evolutionarily very distant from it. We hope this work will contribute to increasing awareness of the importance of developing novel ribosome biogenesis modulators and of preserving natural biodiversity so that natural sources of bioactive compounds can be explored sustainably.

## Materials and methods

All reagents used in this work are listed in Table S1. All original uncropped blots are provided in Fig. S1.

### Human cell cultures

Cells were cultured at 37 °C under 5% CO_2_. HCT116 cells were cultured in McCoy’s medium (Lonza) supplemented with 10% fetal bovine serum (Sigma) and 1% penicillin-streptomycin mix (Lonza). HeLa cells and skin fibroblasts were cultured in DMEM (Lonza) supplemented with 10% fetal bovine serum and 1% penicillin-streptomycin mix. SiHa cells were cultured in EMEM (ATCC) supplemented with 10% fetal bovine serum and 1% penicillin-streptomycin mix. MCF7 cells were cultured in EMEM supplemented with 10% fetal bovine serum, human recombinant insulin (Sigma) at 0.01 mg/mL, and 1% penicillin-streptomycin mix. MDA-MB-231 cells were cultured in DMEM supplemented with 10% fetal bovine serum, 1% MEM non-essential amino acid solution (100x) (Sigma), and 1% penicillin-streptomycin mix.

### Yeast cell cultures and growth assay

The yeast strain BY4741 (purchased from Euroscarf) was cultured at 30 °C in YPD medium (1% w/v yeast extract, 2% w/v peptone, 2% w/v glucose) or minimal medium (0.5% w/v ammonium sulfate, 0.17% w/v yeast nitrogen base, 2% w/v glucose) supplemented with different concentrations of nitidine chloride or BMH-21. For the yeast growth assay, cells were cultured overnight in YPD/minimal medium and serially diluted starting from OD_600_ = 1.0. Five microliters of each diluted culture was spotted onto appropriate plates and incubated at 30 °C for 3 days.

### IC_50_ determination with a resazurin assay

The IC_50_ was determined with a standard resazurin assay: in metabolically active cells, non-fluorescent resazurin is reduced by dehydrogenases to fluorescent resorufin. The level of resorufin fluorescence (Ex = 560 nm; Em = 590 nm) was used as a proxy for cell viability. In practice, 10,000 cells were seeded per well in a 96-well plate (Greiner) and left to adhere overnight. A series of seven concentrations of each tested compound (0.4, 0.8, 1.6, 3.1, 6.2, 12.5, and 25 µg/ml) was applied to plated cells for 48 h, with each concentration tested in triplicate. Next, 20 µl of ready-to-use resazurin solution (Promega) was added to each well. After a 3-h incubation at 37 °C under 5% CO_2_, fluorescence was measured with a microplate reader (Tecan, Infinite M200 PRO). As a control, cells were treated with 0.1% DMSO. The data were analyzed and plotted and the IC_50_ was calculated with GraphPad Prism (9.5.1).

### Clonogenic assay

A total of 1000 Hela or HCT116 cells were seeded into 6-well plates. After attachment, the cells were treated with nitidine chloride for 6 h. Drug-containing medium was removed and the cells were incubated in drug-free medium for 10 days. At the end of the incubation period, the cells were fixed with methanol and colonies were visualized by crystal violet staining.

### Protein translation assay

Click chemistry was used to track newly synthesized proteins and flow cytometry to quantify them. HeLa cells treated for 23 h with nitidine chloride were incubated with L-homopropargylglycine in methionine-free medium for 1 h and global protein translation was determined with the Click-IT™ Plus Alexa Fluor™ 647 Picolyl Azide Toolkit (ThermoFischer Scientific) according to the manufacturer’s instructions, with minor modifications. Briefly, cells were collected after various treatments and incubated with Dead dye (Invitrogen) for 10 min at 4 °C. The cells were washed with PBS, fixed in 4% formaldehyde (Sigma-Aldrich), then permeabilized with Triton X-100 (Sigma-Aldrich) and incubated with Click-IT reaction cocktail for 30 min at room temperature. FACS analyses for quantifying global protein translation versus viable cells were performed with a FACSCanto II flow cytometer and FlowJo software; data were collected from at least 3000 recorded events.

### Western blotting

Total protein was extracted from ~1 × 10^6^ cells. The cells were washed once with ice-cold 1x phosphate-buffered saline (PBS) and then lysed for 15 min at 4 °C in lysis buffer (50 mM Tris-HCl, pH 7.5, 250 mM NaCl, 2 mM EDTA, 0.5% v/v NP-40, 10% v/v glycerol) supplemented with cOmplete Protease Inhibitor Cocktail (Roche Diagnostics). Supernatants were collected by centrifugation at 20,000 × *g* Twenty micrograms of total protein (quantified with the Bradford protein assay dye reagent; Biorad) was loaded onto a polyacrylamide gel containing a separation gel and a stacking gel, resolved, and transferred to a nitrocellulose membrane (Cytiva). The ECL kit (Amersham biosciences) was used for detection. Primary antibodies were used at the following dilutions: anti-p53 (1:1000), anti-beta-actin (1:1000), anti-RPA194 (1:1000). The secondary antibody used were anti-mouse IgG-HRP (1:5000) and anti-rat IgG-HRP (1:2000).

All original uncropped blots are presented in Fig. S1.

### Pre-rRNA processing analysis by northern blotting

HCT116 and HeLa cells were harvested following drug treatment. Total RNA was extracted with TRI reagent solution (Thermofisher) according to the manufacturer’s instructions. For yeast cells, total RNA extraction was performed as described by Sharma et al. [70]. To analyze high-molecular-weight RNAs, 5 μg total RNA from human cells or 8 μg total RNA from yeast cells was resolved in a 1.2% formaldehyde-agarose denaturing gel, followed by electrophoresis at 65 V. Agarose gels were transferred overnight by capillarity onto Hybond-N+ membranes (Cytiva) in 10x saline sodium citrate. Membranes were prehybridized at 65 °C for 1 h in a solution containing 50% formamide, 5x SSPE, 5x Denhardt’s solution, 1% (wt/vol) SDS, and 200 mg/ml fish sperm DNA (Roche). Hybridization with specific ^32^P-labeled oligonucleotide probes was then carried out for 1 h at 65 °C and overnight at 37 °C. Probe sequences are listed in Table S1. The membranes were washed and exposed to Fuji imaging plates (Fujifilm). Signals were captured with a Phosphorimager (FLA-7000, Fujifilm) and quantified with Multi Gauge Software (Fujifilm, v 3.1).

### Pre-rRNA processing analysis by pulse-chase analysis

Pulse-chase metabolic labeling was performed essentially as described in Zorbas et al. [71]. HeLa cells were seeded and treated for 5 h with nitidine chloride, then incubated in methionine-free medium for 30 min, prior to incubation for 30 min with 50 µCi of L-(methyl-^3^H)-methionine. The cells were washed, re-incubated in 10x methionine medium, and harvested at different time points (0, 15, 30, 60, 90, and 120 min). Total RNA was isolated and analyzed as described above except that the membrane was directly exposed to a tritium sensitive plate (Fuji) instead of being probed with radioactively-labeled oligonucleotides.

### Immunofluorescence and imaging of human cells

Immunofluorescence analysis was carried out as described in Nicolas et al. [47]. Briefly, cells were seeded into a 96-well plate (Porvair Sciences) and allowed to adhere overnight. They were treated with the test compound for the appropriate time. After fixation with 4% formaldehyde, they were washed and blocked with 5% BSA and 0.3% Triton X-100 in PBS for 60 min. The primary antibodies used and their respective dilutions in PBS were: anti-fibrillarin 1:1000, anti-PES1 1:500, anti-UBF 1:1000, and anti-γH2AX 1:1000. The appropriate antibodies were added to each well and incubated overnight (except for anti-γH2AX, which was incubated for 1 h only). The following secondary antibodies were used as needed at 1:1000 dilution for 1 h: alexa fluor 594 goat anti-mouse, alexa fluor 488 chicken anti-rabbit, alexa fluor 488 goat anti-mouse IgG, alexa fluor 568 goat anti-rat. For nuclear staining, DAPI was added and incubated for 15 min. Imaging was performed on a Zeiss Axio Observer.Z1 microscope equipped with a 63x/1.4 oil DIC objective (Plan-Apochromat, Zeiss) and controlled by MetaMorph (MDS Analytical Technologies).

### Imaging of GFP-tagged yeast strains

A YLR197W(SIK1/NOP56)-GFP yeast strain grown to mid-log phase in 2% minimal glucose medium (SD) lacking histidine was analyzed in 96-well glass-bottom microscope plates pre-treated with concanavalin A (50 mg/ml). Cells were incubated in SD medium containing 1 mg/ml DAPI as a nuclear marker. Images were captured with a 100x/1.4 oil DIC objective (Plan-Apochromat, Zeiss) on a Zeiss Axio Observer.

### Segmentation and quantification of the yeast fluorescence images

On GFP images converted to 8-bit images, nucleoli were individually segmented with an intensity-based thresholding plugin in Image J (the same threshold was applied to all treatments). For each condition, nucleolar area and intensity were measured from >500 cells across nearly 30 independent images. The data were exported to Microsoft Excel and violin plots were generated with GraphPad Prism. The horizontal lines represent mean values.

### Quantitative and qualitative assessment of nucleolar disruption by iNo scoring

Cells were treated with the indicated drugs or siRNAs (for uL5 and uL18 depletion), processed for imaging, and the images analyzed exactly as described in references [47, 48]. Briefly, five shape and textural parameters of the nucleolus, previously established as the most discriminant ones, were used to cluster nucleolar disruption phenotypes using a principal component analysis. These parameters were: (i) area, (ii) elliptical regularity, (iii) percentage of pixels below an optimized intensity threshold, (iv) lowest intensity, and (v) number of local minima.

For drug treatment: 20,000 HeLa FBL-GFP cells were treated with each drug for 6 h (for NC) or 2 h (for the other compounds) at the concentration indicated in the legend of Fig. S3. For factor depletion: 7000 cells were reverse-transfected with 10 nM siRNAs for 3 d. All treatments were performed in triplicate. Cells were fixed with 4% formaldehyde, washed in PBS, and stained with DAPI (1:20,000 dilution of 5 mg/mL stock in PBS) for 10 min at RT. Imaging was performed on a Zeiss Axio Observer Z1 microscope with a motorized stage, controlled by MetaMorph. Widefield images were captured with a 20x objective (Plan NeoFluar, Zeiss), LED illumination (CoolLed pE-2), and a CoolSnap HQ2 camera. Sixteen independent fields of view were automatically captured for each well, the correct focal plane being maintained by the MetaMorph autofocus module.

### DNA intercalation assays

Two hundred and fifty nanograms of a supercoiled plasmid DNA (pUC19) was incubated with 0.2 μg/ml EthBr at 37 °C for 15 min. This was followed by incubation for 30 min with either 0.1% DMSO (negative control) or NC (1.1 μM, 11 μM, 110 μM, or 1.1 mM) in a reaction buffer containing 10 mM Tris-HCl (pH 7.5), 50 mM NaCl, and 1 mM EDTA. The reaction mixtures were resolved on a 1% agarose gel and DNA was visualized under UV light.

All assays were performed in triplicate (*n* = 3).

### Topoisomerase I and II assays

HeLa extracts purified according to Nitiss et al. [72] were used as sources of topoisomerase activity. Briefly, 10^7^ HeLa cells were harvested and washed in ice-cold phosphate-buffered saline (PBS), to remove residual medium. To release nuclear proteins, the cells were lysed in a buffer containing 20 mM Tris-HCl (pH 7.5), 5 mM KCl, 1 mM MgCl_2_, 10% (v/v) glycerol, 1 mM DTT, and cOmplete^TM^ Protease Inhibitor Cocktail (Roche). The lysate was centrifuged at high speed, and the supernatant was discarded. To enrich in nuclear topoisomerases, the nuclear pellet was subjected to high-salt extraction with 0.35 M KCl in the lysis buffer to release chromatin-associated proteins. The mixture was centrifuged and the supernatant containing the extracted proteins was collected for subsequent assays.

To evaluate NC inhibition of TOP1 and TOP2 activity, in vitro assays were performed with the human topoisomerase I assay kit (TG1015-1, Topogen) and the human topoisomerase II assay kit (TG1001-1, Topogen). Supercoiled plasmid DNA (pHOT1) and kDNA were used, respectively, as substrates for the TOP1 and TOP2 assays.

For the TOP1 assay, 250 ng supercoiled plasmid DNA was incubated with 1 µL HeLa cell extract in TOP1 reaction buffer containing varying concentrations of NC. The final reaction volume was 20 µL and the mixture was incubated at 37 °C for 30 min. Reactions were terminated by adding stop solution and proteinase K. This was followed by incubation at 50 °C for 30 min, to degrade bound proteins.

For the TOP2 assay, 200 ng kDNA was used as a substrate. Reactions contained 1 µL HeLa cell extract, 1 mM ATP, and TOP2 reaction buffer with varying concentrations of NC. The final reaction volume was 20 µL and the mixture was incubated at 37 °C for 30 min. Reactions were stopped by adding stop solution and proteinase K.

Reaction products were analyzed by 1% agarose gel electrophoresis. Gels were stained with ethidium bromide (0.2 µg/ml) and visualized under UV light.

All assays were performed in triplicate (*n* = 3).

### Integrated stress response monitoring

1 × 10^6^ HeLa cells were treated with 0.1% DMSO or 2.2 μM NC or 0.5 μM thapsigargin (ENZO Life Science) for 6 h, then collected for western blot analysis with antibodies specific to the phosphorylated and unphosphorylated forms of eIF2α.

## Supplementary information

Supplementary Information File

## Data Availability

All datasets on which the conclusions of the paper rely are presented in the main manuscript or in additional supporting files. Additional information is available to readers upon request.
