# Supplementary material for: The natural alkaloid nitidine chloride targets RNA polymerase I to inhibit ribosome biogenesis and repress cancer cell growth
Source: Cell Death Discov. 2025 Mar 22;11:116. doi: 10.1038/s41420-025-02396-x (PMC11929923; doi:10.1038/s41420-025-02396-x)
Supplement: Supplementary file 1 — Supplementary Information File [file 41420_2025_2396_MOESM1_ESM.pdf]

SUPPLEMENTARY INFORMATION FILE

For:

"The Natural Alkaloid Nitidine Chloride Targets RNA Polymerase I to Inhibit Ribosome Biogenesis and Repress Cancer Cell Growth"

Igor **VOUKENG**[1,*], Jing **CHEN**[1,*], and Denis L.J. **LAFONTAINE**[1]

[1]RNA Molecular Biology, Fonds de la Recherche Scientifique (F.R.S./FNRS), Université libre de Bruxelles (ULB), Biopark campus, B-6041 Gosselies, Belgium

[*]Equal contribution of two first authors

Table of Contents:

Figure S1: Original uncropped blots

Figure S2: Expanded views of the nucleolar phenotypes presented in Fig 4

Figure S3: iNo scoring assessment of nucleolar disruption caused by NC treatment

Figure S4: Nitidine chloride is a DNA-intercalating agent

Figure S5: Nitidine chloride activates mildly the integrated stress response

Table S1: Reagents used in this study

Related to Figure 4c:

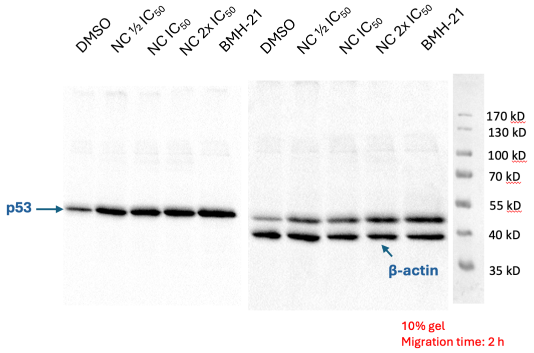

Related to Figure 5b:

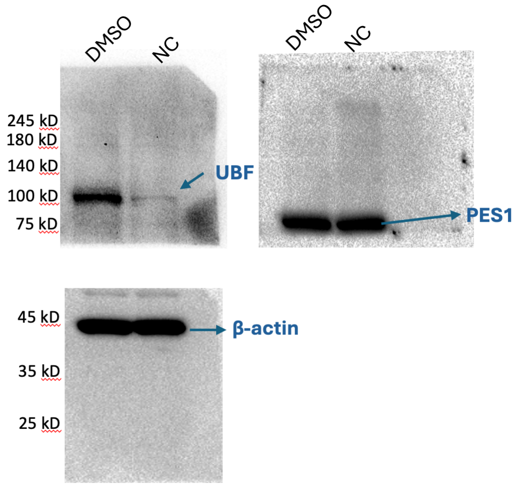

Related to Figure 5c:

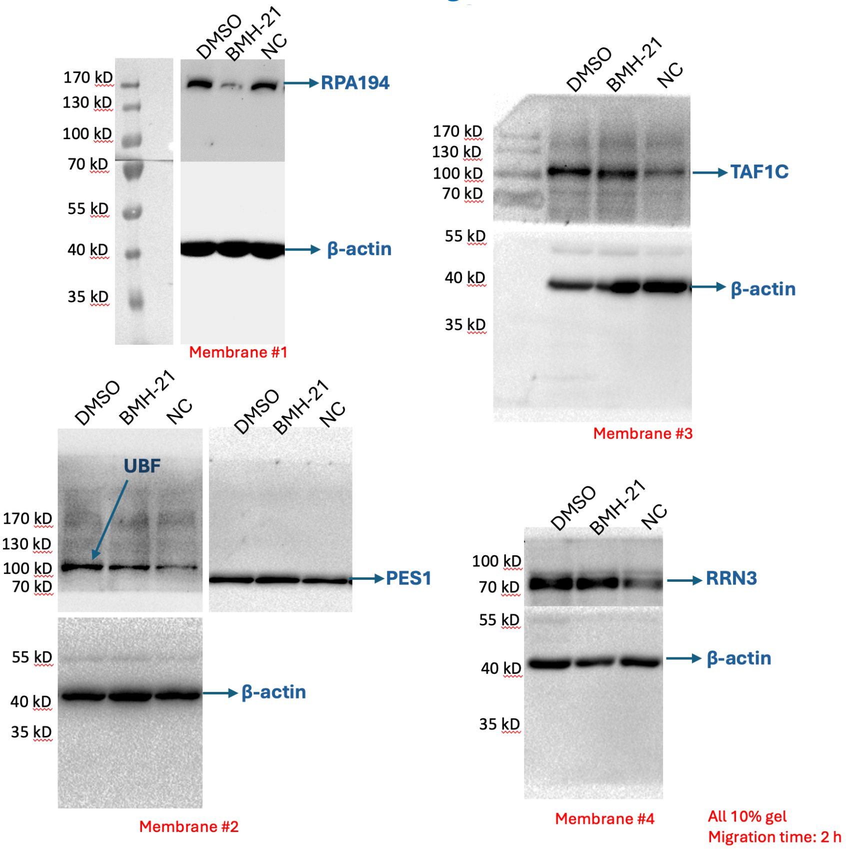

Related to Figure 5d:

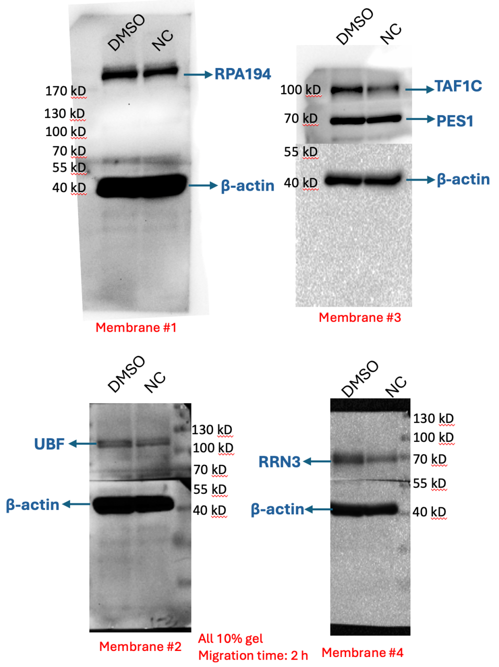

Related to Figure 5e:

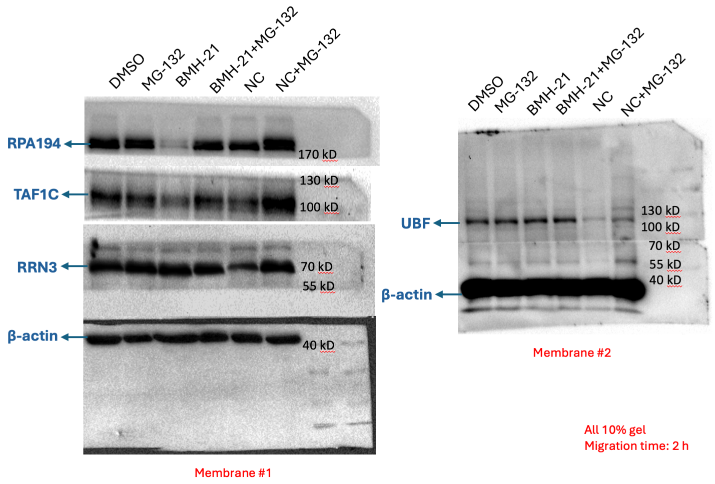

Related to Figure S5:

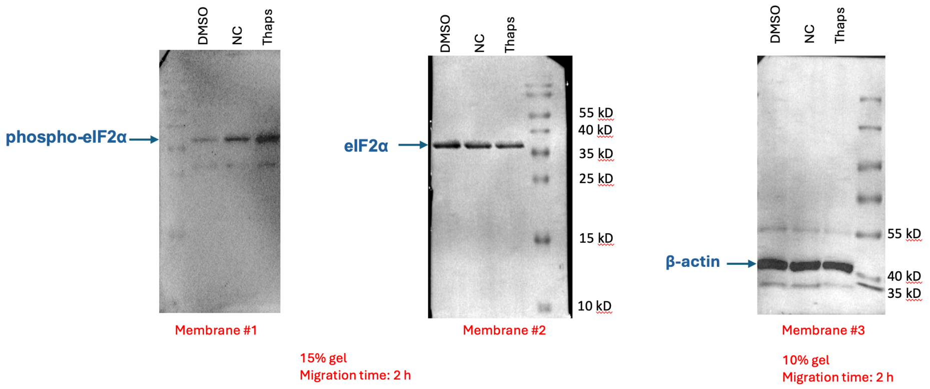

Fig S1: Original uncropped blots

All western blots presented in the manuscript are presented uncropped for reference.

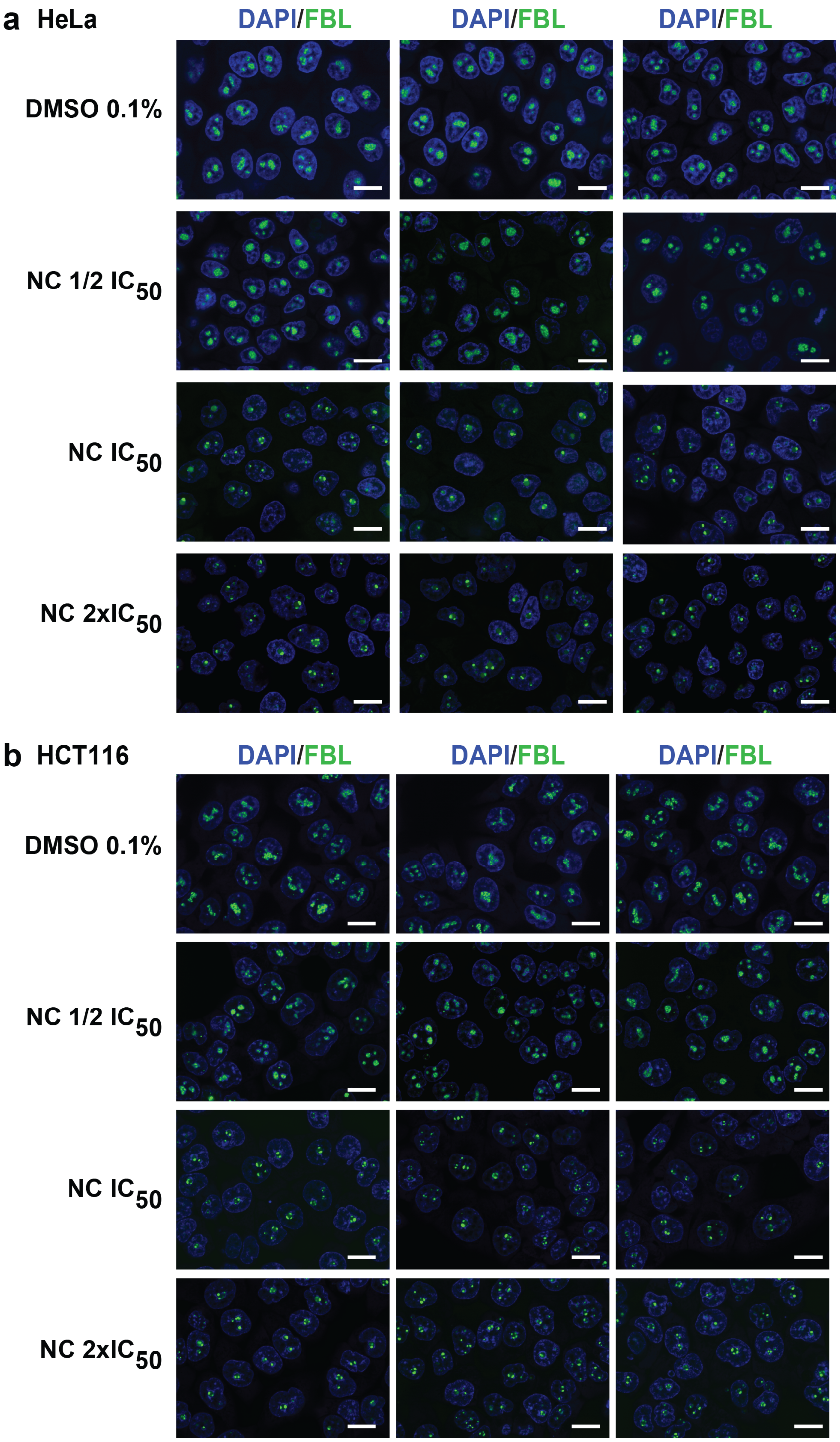

Fig S2: Expanded views of the nucleolar phenotypes presented in Fig 4

The data highlight that the penetrance of nucleolar disruption is near 100%, i.e. that nearly all cells form caps upon treatment with NC at IC50 and above. Three fields of view are shown per condition. Spinning disk, 63x objective. Scale bar, 20 µm.

a, HeLa cells. Same as Fig 4 panel a.

b, HCT116 cells. Same as Fig 4 panel b.

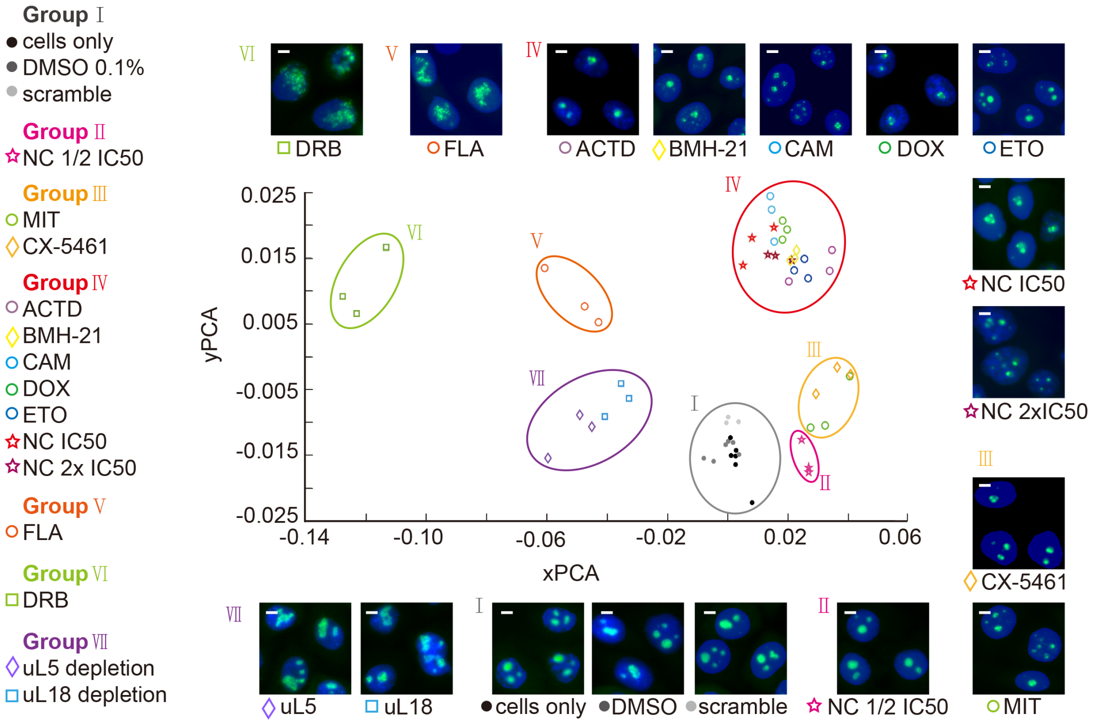

Fig S3: iNo scoring assessment of nucleolar disruption caused by NC treatment

The principal component analysis (PCA) displays the clustering of similar nucleolar disruption phenotypes in a two-dimensional space. Representative images of cells are shown around the PCA graph (green channel, nucleolus/fibrillarin; blue channel, nucleoplasm/DAPI). Scale bar, 5 µm.

HeLa cells expressing a green fluorescent-tagged version of the nucleolar protein fibrillarin (HeLa FBL-GFP) were exposed to the indicated treatments, and images were acquired on a high-throughput platform and analyzed by the iNo scoring method (see Materials and Methods, and [1, 2]). Cells were treated for 6 h with nitidine chloride (NC) at ½ IC_{50} (0.55 µM), IC_{50} (1.1 µM), or 2x IC_{50} (2.2 µM). As controls, cells were treated for 2 h with actinomycin D (ACTD, 0.3 µM), BMH-21 (2 µM), camptothecin (CAM, 25 µM), CX-5461 (10 µM), DRB (100 µM), doxorubicin (DOX, 5 µM), etoposide (ETO, 500 µM), flavopiridol (FLA, 1.2 µM), or mitoxantrone (MIT, 3 µM). For depletion of uL5 or uL18, cells were transfected with siRNAs targeting their respective mRNAs (10 nM, 3 days). Controls also included untreated cells (cells only), cells treated with DMSO (0.1%), and cells treated with a non-targeting silencer (scramble).

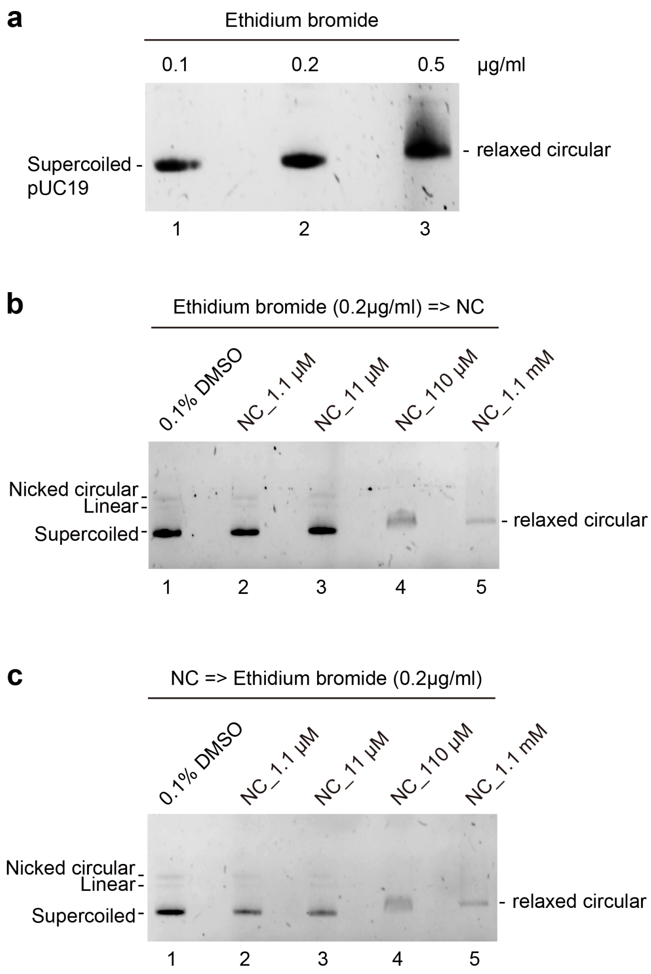

Fig S4: Nitidine chloride is a DNA-intercalating agent

A supercoiled DNA (pUC19) was incubated with ethidium bromide (EthBr), nitidine chloride (NC), or DMSO (used as control) at the indicated concentrations, separated by 1% agarose gel electrophoresis, and visualized under UV light.

a, Incubation of DNA with ethidium bromide alone reveals that a high concentration (0.5 µg/ml) of the compound shifts the supercoiled plasmid to a slower-migrating relaxed circular form. The DNA is simply stained at lower concentrations (0.1 or 0.2 µg/ml).

b, Incubation of DNA for 15 min with EthBr at a concentration that does not relax the plasmid (0.2 µg/ml) was followed by incubation for 30 min with increasing concentrations of NC (a 1.1 µM-to-1.1 mM range was tested). This revealed that at 110 µM NC and above the DNA is relaxed and the intensity of fluorescent staining is decreased. This indicates that NC is a DNA-intercalating agent which has partly chased EthBr.

c, Same experiment as presented in **b** with the order of incubations reversed (NC first and then EthBr) and reaching the same conclusions.

All assays were performed in triplicate (n = 3).

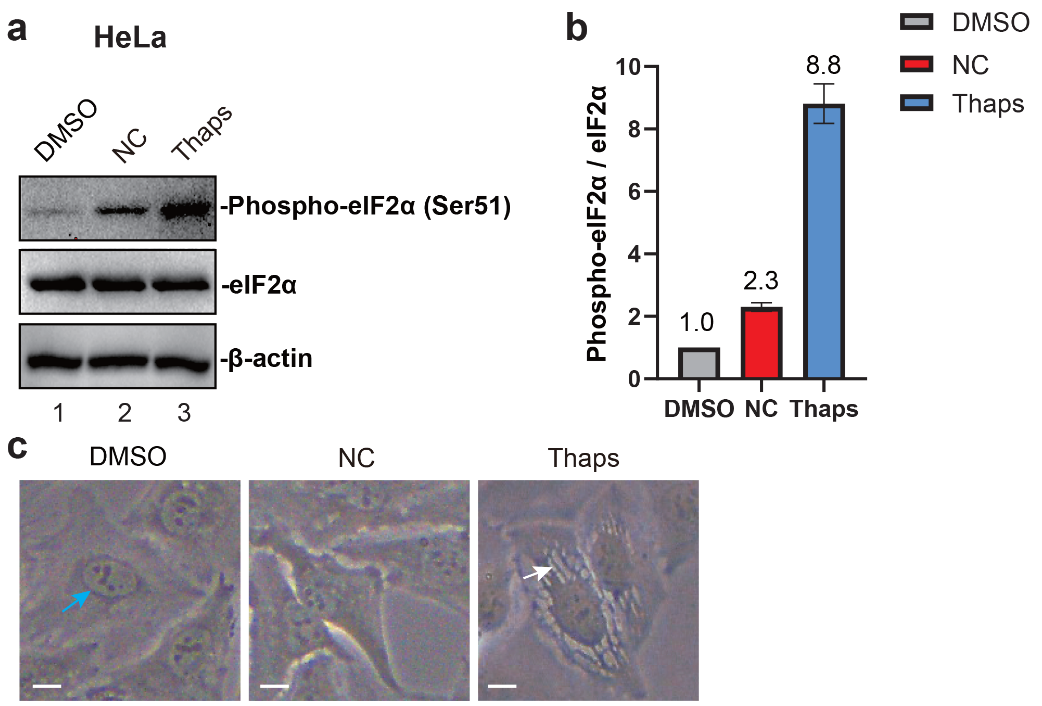

Fig S5: Nitidine chloride activates mildly the integrated stress response

a, Integrated stress response activation monitored by detection of phosphorylated eIF2α. Total protein extracted from HeLa cells treated with nitidine chloride (NC, 2.2 μM), thapsigargin (Thaps, 0.5 μM), or DMSO (0.1%) for 6 h were analysed by Western blotting with specific antibodies against eIF2α, or Phospho-eIF2α (Ser51). As loading control, β-actin was used.

b, Quantification of panel **a**. Data presented as means of three independent experiments (n= 3).

c, Microscopic inspection of HeLa cells treated with the indicated drugs, as in panel a. In the DMSO panel, the blue arrow points to a nucleus (with nucleoli visible inside as intense dark foci). In the Thaps panel, the white arrow points to remarkable cytoplasmic structures forming upon ISR activation. Bright field observation with 40x objective. Scale bar, 5 μm.

TABLE S1: Reagents used in this study

Resources	Origin	Reference
**Cell lines**		
HeLa	ATCC	CCL-2
HeLa FBL-GFP	Lafontaine Lab	Ref. [1]
HCT116 p53+/+	ATCC	VVL-47
HCT116 p53-/-		Ref.[3]
SiHa	ATCC	HTB-35
MCF7	ATCC	HTB-22
MDA-MB-231	ATCC	HTB-26
Skin fibroblasts, healthy	Kind gift from Dr Diane Doummar (APHP, Paris)	
**Yeast strains**		
BY4741	Euroscarf	Y0000
YLR197W(NOP56/SIK1)-GFP	Euroscarf	Ref.[4]
**Antibodies**		
anti-phospho-histone γH2AX (Ser139), clone JBW301	Merck	05-636
anti-fibrillarin antibody	Abcam	AB5821
anti-UBF (F-9)	Santa Cruz	SC13125
anti-TIF1C	Santa Cruz	SC374551
anti-p53	Santa Cruz	SC-126
anti-beta actin (AC-15)	Santa Cruz	SC-69879
anti-PES1	IMI Munich	
anti-RPA194	Santa Cruz	SC-48385
anti-eIF2α	Santa Cruz	SC-133132
anti-EIF2S1(phospho Ser51)	Abcam	Ab32157
anti-mouse IgG-HRP	Jackson ImmunoResearch Labs	115-036-062
anti-rat IgH-HRP	Santa Cruz	SC2303
alexa fluor 594 goat anti-mouse	Invitrogen	A-11005
alexa fluor 488 chicken anti-rabbit	Invitrogen	A-21441
alexa fluor 488 goat anti-mouse IgG	Invitrogen	A-11001
alexa fluor 568 goat anti-rat	Invitrogen	A-11077
**Chemicals & Reagents**		
TRI reagent	Thermofisher	AM9738
DMEM	Lonza	BE12-604F
McCoy's	Lonza	BE12-688F

fetal bovine serum (FBS)	Sigma-Aldrich	F7524
penicillin-streptomycin	Lonza	DE17-602E
MEM non-essential amino acid solution (100x)	Sigma	M7145
L-glutamine	Lonza	BE17-605E
DPBS	Lonza	17-516 F
trypsin/EDTA solution	Lonza	CC-5012
concanavalin A	ThermoFisher	J61221.MC
cell titer-Blue®	Promega	G8080
click-IT™ plus alexa fluor™ 647 picolyl azide toolkit	ThermoFischer Scientific	C10643
DMSO	Sigma-Aldrich	D265
BMH-21	Sigma-Aldrich	SML1183
5-fluorouracil	Sigma-Aldrich	F6627
CHX (cycloheximide)	CarboSynth	AC05909
NC (nitidine chloride)	Sigma-Aldrich	SML0610
MG-132	Sigma-Aldrich	M7449
Thapsigargin	ENZO Life Science	ENZOBMLPE1800001
DRB (5,6-dichloro-1-β-D-ribofuranosylbenzimidazole)	CarboSynth	ND06701
FLA (flavopiridol)	Sigma	F3055
ACTD (actinomycin D)	Sigma	A4262
CAM (camptothecin)	CarboSynth	FC15450
DOX (doxorubicin)	CarboSynth	AD15377
ETO (etoposide)	CarboSynth	ME09941
CX-5461	Sigma	509265
MIT (mitoxantrone)	CarboSynth	FM61451
Hybond N+ membrane	Cytiva	RPN203B
Topoisomerase I assay kit	Topogen	TG1015-1
Topoisomerase II assay kit	Topogen	TG1001-1
**Oligonucleotides (northern blot probes)**		
Human probes		
5’ETS probe (LD1844): 5’- CGGAGGCCCAACCTCTCCGACGACAGGTCGCCAGAGGACAGCGTGTCAGC-3’		
ITS1 probe (LD1827): 5’- CCTCGCCCTCCGGGCTCCGTTAATGATC-3’		
ITS2 probe (LD1828): 5’- CTGCGAGGGAACCCCCAGCCGCGCA-3’		
Yeast probes		
ITS2 E-C2 (LD0339): 5’-GGCCAGCAATTTCAAGTTA -3’		
ITS1 A2-A3 probe (LD0359): 5’- TTGTTACCTCTGGGCCC-3’		
ITS1 D-A2 probe (LD0471): 5’- CGGTTTTAATTGTCCTA -3’		

siRNAs	
SCR (LD234)	rCrGrUrUrArArUrCrGrCrGrUrArUrArArUrArCrGrCrGrUAT
	rArUrArCrGrCrGrUrArUrUrArUrArCrGrCrGrArUrUrArArCrGrArC
uL5 (LD695):	rArUrArUrGrArCrCrCrArArGrCrArUrUrGrGrUrArUrCrUAC
	rGrUrA rGrArU rArCrC rArArU rGrCrU rUrGrG rGrUrC rArUrA rUrUrU
uL18 (LD698):	rCrGrC rUrUrG rGrUrG rArUrA rCrArA rGrArU rArArA rArAT A
	rUrArU rUrUrU rUrArU rCrUrU rGrUrA rUrCrA rCrCrA rArGrC rGrUrU

Softwares	
FlowJo	BD bioscience
Image J	National Institutes of Health, USA<br>http://imagej.nih.gov/ij
Image Lab v 6.0.1	Bio-RAD Laboratories
FLA	Fujifilm
MultiGauge v 3.1	Fujifilm
Graphpad Prism v 9.5.1	https://www.graphpad.com/
Metamorph®	MDS Analytical Technologies
AutoDock Tool 1.5.7	https://ccsb.scripps.edu/mgltools
Autodock Vina 1.2.2	https://vina.scripps.edu/
PyMOL 3.0.3	https://www.pymol.org/

Equipment		
FACS Canto II flow cytometer	BD bioscience	N/A
Axio Observer Z1	Zeiss	N/A
pE-2 LED light source	CoolLed	N/A
Spinning disk confocal head	Yokogawa	N/A
CCD camera	HQ2	N/A
20x (0.5 NA) EC Plan Neofluar	Zeiss	N/A
40x (0.75 NA) EC Plan Neofluar	Zeiss	N/A
63x/1.4 oil DIC Plan-Apochromat	Zeiss	N/A
100x/1.4 oil DIC Plan-Apochromat	Zeiss	N/A
Infinite M200 PRO	Tecan	N/A

REFERENCES TO SUPPLEMENTARY INFORMATION FILE

1. Nicolas E, Parisot P, Pinto-Monteiro C, de Walque R, De Vleeschouwer C, Lafontaine DLJ. Involvement of human ribosomal proteins in nucleolar structure and p53-dependent nucleolar stress. *Nature communications* 2016, **7:** 11390.

2. Stamatopoulou V, Parisot P, De Vleeschouwer C, Lafontaine DLJ. Use of the iNo score to discriminate normal from altered nucleolar morphology, with applications in basic cell biology and potential in human disease diagnostics. *Nature protocols* 2018, **13**(10)**:** 2387-2406.

3. Tafforeau L, Zorbas C, Langhendries JL, Mullineux ST, Stamatopoulou V, Mullier R*, et al.* The complexity of human ribosome biogenesis revealed by systematic nucleolar screening of Pre-rRNA processing factors. *Molecular cell* 2013, **51**(4)**:** 539-551.

4. Huh WK, Falvo JV, Gerke LC, Carroll AS, Howson RW, Weissman JS*, et al.* Global analysis of protein localization in budding yeast. *Nature* 2003, **425**(6959)**:** 686-691.
